# Attachment of Hydrogel Patches to Eye Tissue through Gel Transfer using Flexible Foils

**DOI:** 10.1021/acsami.4c15089

**Published:** 2025-01-28

**Authors:** Shubham Tiwari, Luisa Goldmann, Jan Lübke, Oswald Prucker, Gottfried Martin, Günther Schlunck, Jürgen Rühe

**Affiliations:** † Department of Microsystems Engineering (IMTEK), Laboratory for Chemistry & Physics of Interfaces (CPI), Albert Ludwigs Universität Freiburg, Georges Köhler Allee 103, 79110 Freiburg, Germany; ‡ Cluster of Excellence livMatS @ FIT−Freiburg Center of Interactive Materials and Bioinspired Technologies Albert Ludwigs Universität Freiburg, Georges Köhler Allee 105, 79110 Freiburg, Germany; § Eye Center, Medical Center − University of Freiburg, Faculty of Medicine, University of Freiburg, Killianstraße 5, 79106 Freiburg, Germany

**Keywords:** C−H insertion cross-linking (CHic), Glaucoma, Surface-attached polymer network, Protein-repellent hydrogel, TUNEL staining, Histone2A staining

## Abstract

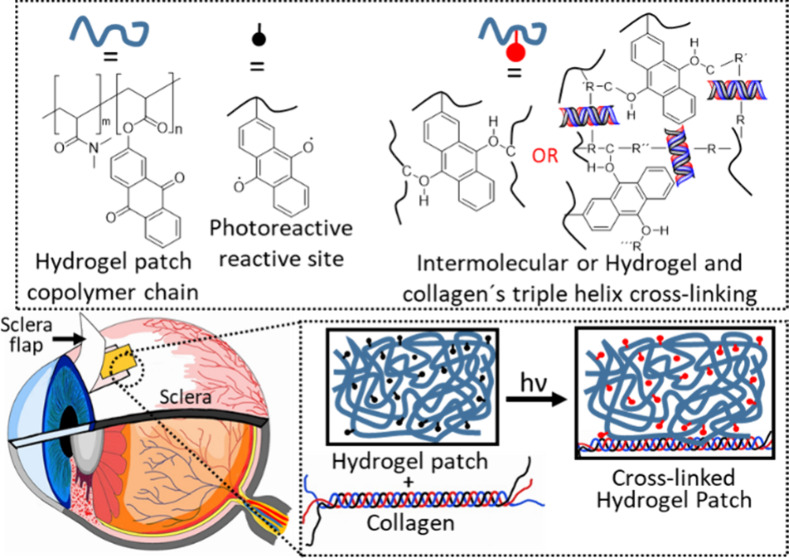

Glaucoma, a leading cause of blindness, demands innovative and effective treatments that surpass the limitations of current drug and surgical interventions to lower intraocular pressure. This study describes the generation of cell-repellent hydrogel patches, their deposition on the ocular surface, and a photoinduced chemical binding between the patches and the collagens of the eye. The hydrophilic and protein-repellent hydrogel patch is composed of a copolymer made from dimethylacrylamide and a comonomer unit with anthraquinone moieties. A thin layer of the prepolymer is deposited on a flexible foil. This foil has a hydrophobic side on top and superhydrophilic sides on the bottom. A small amount of added water can penetrate easily through the foil and release the polymer, which is thus transferred from the foil onto the eye at the desired location. Through brief irradiation with UV light with a wavelength of 365 nm, the prepolymer is simultaneously cross-linked and covalently attached to the eye’s surface through C–H insertion reactions. We describe the handling, attachment, UV irradiation effects on nearby healthy tissues, biocompatibility, stability, and cellular interaction of the patches during in vitro studies.

## 1 Introduction

Glaucoma is a severe, chronic eye disease and a leading cause of irreversible blindness worldwide.^1,2^ It primarily stems from too high intraocular pressure (IOP), exerting pressure on the optic nerve, which is crucial for transmitting visual information from the retina to the brain.^2−4^ Constant high pressure for a long period of time causes damage of the delicate fibers of the optical nerve and leads to irreversible vision loss.^5^ It is the leading cause of blindness, affecting about 80 million people globally,^6^ many of whom are unaware of their condition.^6^ The WHO projects glaucoma cases to reach 111.8 million by 2040, highlighting the urgent need for effective treatments.^7−15^

Current glaucoma treatments involve improving the flow of aqueous humor via medication and laser therapy. In severe cases, the trabecular meshwork is partially removed, or an artificial filtering system is surgically implanted to create an outflow through the sclera to the sub conjunctival layer.^16^ However, these approaches often fail within 3–4 months due to episcleral or sub conjunctival scar tissue formation by Tenon fibroblast cells.^17−20^ One trigger for postoperative scarring is the intraoperative collapse of the blood-aqueous barrier. This results in the release of a variety of proinflammatory cytokines, which stimulate scar tissue formation.^21^ To reduce scar formation, drugs like Mitomycin C or 5-fluorouracil, are introduced during the surgery, but they increase the risk of infection and damage healthy tissue.^16,22−25^ Additionally, tube implants, which are designed to maintain humor flow, can be blocked by fibroblasts, resulting in treatment failure and necessitating additional surgeries.^26−28^

A general approach to reduce scarring is to prevent the adhesion of scar-forming cells, namely Tenon fibroblast cells, in the case of the sclera of the eye. An important strategy to reduce cellular adhesion permanently is to make the surfaces protein-repellent and thus bioinert. A common way to reduce protein adhesion is to reduce the surface tension of the surface in contact with the proteins, especially by using fluorinated materials.^22−28^ However, such an approach will strongly influence the wetting of these surfaces and is thus not really suitable for eye surgery.^29,30^

It has been shown that surface-attached neutral hydrogels, which do not show any enthalpic interactions (i.e., no Coulombic or hydrophobic interactions) with proteins, do not show any unspecific protein adsorption.^42,45^ As for the incorporation of proteins to a swollen polymer layer, the mixing entropy is small; the addition of the proteins to the polymer leads to an entropy loss, making protein adsorption energetically unfavorable (entropic shielding).^42^ Additionally, the size exclusion effect prevents larger proteins from penetrating the gel. These properties render the material bioinert, suitable even for blood-compatible coatings.^32,42−45^

In a previous study, we reported on an approach to prevent Tenon fibroblast cells from adhering to scleral tissue by using surface-attached protein-repellent hydrogel pads.^42^ The chosen material was a hydrogel copolymer patch prepared from a precursor polymer consisting of 95% dimethyl acrylamide (DMAA) as the hydrophilic moiety and 5% 2-acryloyloxy anthraquinone (AOAQ) as the photoreactive group.^42^ Light-induced activation of the AOAQ groups at 365 nm causes rapid hydrogen abstraction and free radical recombination, linking polymer chains to a network through C–H insertion cross-linking reactions (CHic) and simultaneously the forming hydrogel to the tissue.^46,31^ These hydrogel patches form a protein-repellent hydrogel network, which is covalently attached to the collagen of the ocular tissue using C–H insertion cross-linking (CHic).^31−36^ The process eliminates the need for reactive additives, harsh conditions, or additional reaction and purification steps^31−34^ and provides an excellent adhesion in the presence of water (“under-water adhesion”), making it ideal for such biomedical applications.^31,37−42^

**Figure 1:**
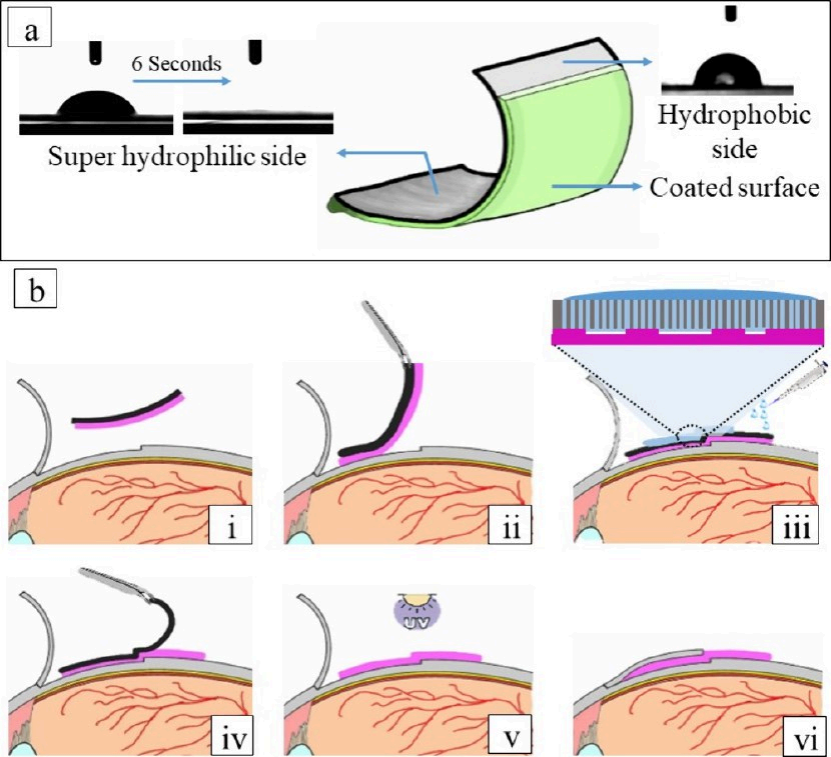
a) Schematic representation of a hydrogel patch carrier. One side of the silanized paper is coated to be hydrophobic with a contact angle of ∼90°, while the opposite side is superhydrophilic with a variation in contact angle from ∼27° to 0° in 6 s. b) Scheme of the application process: (i) patch is directed toward the surface (ii) the flexible paper is used to place the patch on the eye, (iii) hydration of the patch with water allows uniform water distribution on and through the paper, resulting in the release of the patch, (iv) paper is peeled away, (v) patch is cross-linked with UV light. (vi) Finally, the patch is stitched to the flap, ensuring the prevention of scar formation at scleral flap.

As most trabeculectomy procedures are performed with the patient awake, minimal transfer and cross-linking times are required. The process requirements of surgery set the upper limit for the patch transfer and fixation to around 5 min. Typically, for transfer, a sacrificial layer is used (a thin layer connecting the patch and a solid support), which is dissolved once the layer is in place.^42^ However, rigid supports are not easily used in surgery on the curved ocular surfaces. In addition, as the assembly is relatively large, water penetrates slowly from the sides, making the release and transfer process slower. As a result, de-adhesion, and therefore transfer, varies between different users during patch delivery. The aim of the research described in the following is to improve hydrogel patch deposition, release, and fixation by introducing a transfer printing process for delivery of the hydrogel patch. To this, we use a flexible foil, which is based on a paper with dual properties, where one side is hydrophobic and the other superhydrophilic (Figure 1(a)). We use porcine sclera to study the transfer properties and the photoattachment under conditions that mimic the actual trabeculectomy surgery. We optimize the in vitro studies materials and the cross-linking techniques to ensure efficient and fast patch application and secure adhesion.

## 2 Experimental Section

### 2.1 Materials

All chemicals and solvents (reagent grade) were obtained from Merck (Darmstadt, Germany), Carl Roth (Karlsruhe, Germany), VWR (Darmstadt, Germany), SAV Liquid Production GmbH (Flintsbach, Germany), and used as received unless otherwise noted. All water used throughout the experiments was deionized unless stated otherwise. Poly(methyl methacrylate) (PMMA) slides with edges ground, 45°, were commercially purchased from Carl Roth (Germany) and cleaned for 3 min in methanol before usage. Cell culture essentials were purchased from Thermo Fisher Scientific (Germany), Biozol Diagnostica Vertrieb GmbH (Eching, Germany), Jackson ImmunoResearch (Pennsylvania, USA), Promega Biotechnology GmbH (Walldorf, Germany), Cell Signaling Technology (Massachusetts, USA), or Bio&Sell (Feucht, Germany).

### 2.2 Copolymer Synthesis

To incorporate the photo cross-linker 2-acryloyloxy anthraquinone 2-AOAQ (278 g/mol, 0.278 g, 1 equiv) into a copolymer, free radical polymerization was performed with N,N-dimethyl acrylamide (DMAA) (99.13 g/mol, 1.88 g, 19 equiv) as the comonomer and 2,2′-azobis(2,4-dimethyl-4-methoxyvaleronitrile) AMDVN V-70 (308.22 g/mol, 0.058 mmol) as the initiator in N,N-dimethylformamide (DMF) (122.7 g/mol, 25 mL). The resulting copolymer was purified and precipitated in cold diethyl ether, then dried under vacuum overnight. A detailed version of the synthesis protocol is available in Supporting Information. The cross-linker content was determined by ^1^H and ^13^C NMR. ^1^H NMR spectra of the monomer and the copolymer are available in Supporting Information (Figure S3 and S4). The kinetics of activation of the crosslinker were analyzed via UV–vis spectroscopy (not shown), and swelling behavior and protein repellency were assessed using surface plasmon resonance (SPR).

### 2.3 Patch Preparation

A multilayer system was prepared using a microscopic glass slide, double-sided tape as a temporary attachment to hold the paper to the glass slide, and single-sided silanized paper (Figure 1a). The hydrophilic side of the paper was placed against the tape, with the hydrophobic side on top. A 150 mg/mL copolymer solution in ethanol was applied using dip coating, resulting in an approximate dry thickness of 450 nm. The thickness varies from top to bottom, so only the middle area was selected for patch generation (Figure 1b).

### 2.4 UV Illumination Device

A high-performance UV-LED Solo Pen (Optsytec Dr. Gröbel, Ettlingen, Germany) was used as a point light source for manual irradiation. The UV-LED operates at a wavelength of 365 nm. It functions without an external control unit and can be powered directly using a plug-in power supply or controlled via software. The used UV lamp delivers a dose of 200 mW/cm^2^ at full intensity, which decreases with lower intensity settings like 132 mW/cm^2^ at 50%, 62 mW/cm^2^ at 20%, 31 mW/cm^2^ at 10%, and 17 mW/cm^2^ at 5% respectively, measured with a Radiometer RM-22 version 1.03SD (Optsytec Dr. Gröbel, Ettlingen, Germany).

### 2.5 Porcine Sclera

Fresh pig eyes were obtained from a local provider within 4 h post-mortem. The fat tissues and conjunctiva were removed. Depending on the experiment, the eyes were either sectioned into smaller pieces or used whole for patch attachment testing.

### 2.6 Fluorescence Microscopy

The sclera tissue and hydrogel patch was fixed in 4% formalin and dehydrated with ethanol to xylol overnight before embedding in paraffin. 5–10 μm sections were cut and embedded in a fluorescent mounting medium or Entellan. These sections were analyzed using differential interference contrast (DIC), UV channel (365 nm excitation, > 400 nm emission), or TRITC channel (545 nm excitation, 610 nm emission).

### 2.7 Cell Culture

Human Tenon Fibroblasts (HTFs) were sourced from the Department of Ophthalmology, University of Freiburg, Germany, with patient consent. The cells were cultured in DMEM medium supplemented with 10% FCS and penicillin-streptomycin on a T-75 cell culture flask and incubated at 37 °C in 5% CO_2_ (HeraCell 240i, Thermo Fisher Scientific) and then cultivated the cells for 24 h.

### 2.8 Coverslip Preparation

The 13 mm diameter coverslips (Thermanox Plastic coverslips, NUNC Brand products, LOT 1166884) were incubated in 5% KOH in methanol for 30 min, extensively washed with ultrapure water, and stored in 60% isopropanol. The coverslips were dried before use. Coverslips were utilized due to their significant advantage over standard cell wells as they allow for observing cells from the bottom with a fixed focal plane, indicating that cell repellency occurs only at the edges. Additionally, coverslips can be easily flipped upside down to examine the top surface.

### 2.9 Histone2A Staining of Cultured Cells

The cells were cultured in cell-culture plates for 24 h, then transferred to coverslips and incubated for an additional 24 h. The cells on the coverslips were subsequently illuminated with varying doses and time intervals. Treated coverslips with cells were fixed in 4% PFA for 15 min at room temperature (RT). They were washed with DPBS 3–4 times for 5 min each. The samples were incubated in a blocking solution of 5% normal goat serum and 0.3% Triton X-100 in PBS for 1 h at RT. Then, we applied the primary antibody H2A (Antiphospho Histone antibody) (Cell Signaling Technology, USA, catalog no. 9718) in 0.3% Triton X-100 in PBS and incubated the sample at 4 °C overnight. Next, the samples were incubated with the secondary antibody, goat antirabbit, and fluorescent dye Phalloidin TRITC in 0.3% Triton X-100 in PBS for 1 h. After washing with DPBS for 5 min, five times, the samples were mounted in an aqueous mounting medium such as DAPI-Vectashield.

### 2.10 TUNEL (TdT-Mediated dUTP-Biotin Nick End Labeling) Staining of the HTF Cells

The cells were cultured in cell-culture plates for 24 h, then transferred to coverslips and incubated for an additional 24 h. After this the cells on the coverslips were illuminated with varying doses and time intervals. Then, the coverslips were immersed in 4% formaldehyde in PBS buffer for 25 min at 4 °C. The slide was washed twice in PBS for 5 min, then immersed in 0.2% Triton X-100 in PBS for 5 min, then washed again in PBS for 5 min, twice. 100 μL of equilibration buffer was added at room temperature and incubated for 5–10 min. 50 μL of TdT reaction mix was applied to the cells on an area no larger than 5 cm^2^, ensuring the cells would not dry completely. The coverslips were covered for even mix distribution and incubated for 60 min at 37 °C in a humidified chamber, avoiding light exposure. Remove the coverslip, immerse the slide in 2X SSC for 15 min, and then wash in PBS for 5 min, three times. Finally, a mounting medium, such as Vectashield with DAPI, should be added for visualization. Fluorescence microscopy was used to detect localized green fluorescence indicating apoptotic cells, while DAPI-stained nuclei appeared blue.

### 2.11 Surface Plasmon Resonance (SPR)

The layer thickness and the kinetic study of the copolymers were obtained by SPR (modified Res-Tec R2005HT, Res-Tec, Germany) using a setup in the Kretschmann configuration with a He–Ne laser (λ = 632.8 nm), 90° prism (BK7, nD = 1.5151, Spindler & Hoyer, and LaSF9N, n_D_ = 1.8449, Berliner Glas), and index match liquid (n_D_ = 1.5160 and 1.700, Cargill). All reflectivity curves were recorded using p-polarized light. The dry thickness of surface-attached polymer monolayers was measured by ellipsometry (SENTECH Instruments GmbH, Berlin, Germany, incidence angle: 70°, Model: SE 400 adv) on a silanized silicon wafer of square shape sized 15 mm. All the thickness of the coating was calculated with the plasmon stimulation program named WinSpall version 3.02.

### 2.12 Critical Point Drying (CPD)

Critical Point Dryer from Leica Microsystems (Model: EM CPD300) was used to remove moisture from sclera tissue while preserving the morphology of the swollen hydrogel on top. A section of the sclera with the hydrogel patch was placed in a metal mesh. Ethanol was added to achieve sufficient height according to protocol. The sample components reached the critical point, transitioning from liquid to gaseous phases without crossing interfaces, thus avoiding damage. Liquid CO_2_ was used to replace water, as its critical point is at 31 °C and 74 bar, preventing damage to biological samples.

### 2.13 Microcomputed Tomography (Micro-CT)

The gradient hydrogels were cut into pieces and then scanned using a high-resolution micro-CT system (Bruker Skyscan 1272, Kontich, Belgium). The X-ray beam was produced by exposing the tungsten anode to an electron beam of 50 kV. A series of 2D X-ray images of a sample were taken by rotating the sample over 360° with a rotation step of 0.6°. The spatial resolution of the images was kept in a range of 3–5 μm in terms of pixel size. The 3D image of the objects was reconstructed using a modified Feldkamp algorithm for cone-beam acquisition geometry realized in the InstaRecon software. The alignment, beam-hardening, and ring artifacts were corrected before starting the reconstruction process. 3D visualization was done using the Bruker CTVox software.

### 2.14 Scanning Electron Microscopy (SEM)

After critical point drying, the dried tissue was cryo-sectioned to achieve thin slices. The sclera was first cooled with liquid nitrogen and then processed in a cryotome for thin sectioning. The cross sections were polished by the ion beam and coated with gold, where the samples were imaged by a focused ion beam scanning electron microscope (FIB-SEM) (Scios DualBeam) (Quanta FEG 250) under high vacuum conditions. The image was positioned 100 μm below the surface.

## 3 Results and Discussion

### 3.1 Patch Generation and Transfer

Quick and efficient release of the hydrogel patch during the experiment is a crucial step. To enable rapid and reliable transfer of the patch to the eye, a one-sided silanized paper was used as a flexible film to transfer the hydrogel patch. This type of paper is often used to transfer temporary tattoos onto a person’s skin (“tattoo paper”). One side of the paper is hydrophobic, while the other side is super hydrophilic. When water is applied to the hydrophilic side, the water quickly and evenly penetrates the paper. (Figure 1a). To allow the coating to be applied to the hydrophobic side only, the hydrophilic side of the paper is temporarily attached to a rigid support using double-sided adhesive tape. This assembly was dip-coated with the copolymer solution (150 mg/mL) to achieve the desired thickness of 450 nm. Such a thickness of the final patch gives the hydrogel sufficient mechanical stability during transfer but does not have a haptic effect on the patient. A patch of the desired size was cut from this assembly and peeled off the temporary backing. The paper with the hydrogel patch was placed on the substrate, i.e., porcine sclera, with the hydrogel side facing the porcine sclera and the superhydrophilic side facing the user, as shown in Figure 1b. Since, the hydrogel copolymer coating was placed on the hydrophobic side, so the work of adhesion W_A_ between two interfaces can be described as,
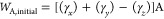
1where *γ*_*hydrogel*_ (*γ*_*x*_) is the surface energy of the hydrogel and *γ*_*hydrophobic surface*_ (*γ*_*y*_) is the surface energy of hydrophobic surface to which it is attached, *γ*_*hydrogel-hydrophobic*_ (*γ*_*z*_) is the interfacial energy between both the material layers and A is the respective area. When a drop of water is applied to the paper backing, it spreads uniformly across the surface, allowing water penetration from the entire back surface. During this water imbibition, the hydrogel patch is released. This changes the interfacial energies as the water interacts with both hydrogel (*γ*_*w*__–*x*_) and the hydrophobic surface(*γ*_*w*__–*y*_):
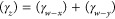
2This leads to the work of adhesion W_A,with water_ in the presence of water:
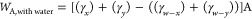
3Therefore, the reduction in the work of adhesion can be written as,
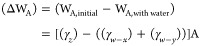
4

In the dry state, the interactions between hydrogel and hydrophobic surface interaction based on the van der Waals forces are moderately strong. In contrast, in the wet state, hydrogel–water interactions based on hydrogen bonding and other dipole–dipole interactions dominate over the interactions between the hydrogel and the nonpolar hydrophobic surface. Because the combined interfacial energies in the wet state are lower than in the dry state, therefore, the change in work of adhesion becomes negative upon contact with water (eq 4). This leads to the release of the gel, facilitating the easy and efficient transfer of the hydrogel patch onto the target substrate (Figure 1(b (iii))). The transferred layer has no damaged edges or shows complete transfer, which is crucial for maintaining the integrity of the patch.^47^ This method ensures uniformity, minimal transfer times, and the ability to reposition the patch after first contact as required. Given the spherical curvature of the eye and the flexible nature of the backing, the paper, with the patch on top, can be gently pressed onto the ocular surface to remove any air bubbles and gently adjusted as required. Another significant advantage of using silanized paper as a transfer foil in scleral treatment is the ability to easily adjust the patch size and location to meet specific needs. This flexibility allows for multiple patches to be created from the same slide, ensuring customized treatment and efficient use of materials.

Once the excess water has been removed, the flexible backing paper can be peeled off, and the hydrogel patch can be touched up with a swab and then photochemically cross-linked using UV light with a wavelength of 365 nm. The UV light is here provided from a pen-shaped source as the focused beam precisely targets the application area. Additionally, it is small and portable, which makes it highly suitable for biomedical and surgical applications. It allows for easy maneuverability and precise control, ensuring efficient and accurate cross-linking with minimal discomfort.

### 3.2 Hydrogel Characterization

**Figure 2:**
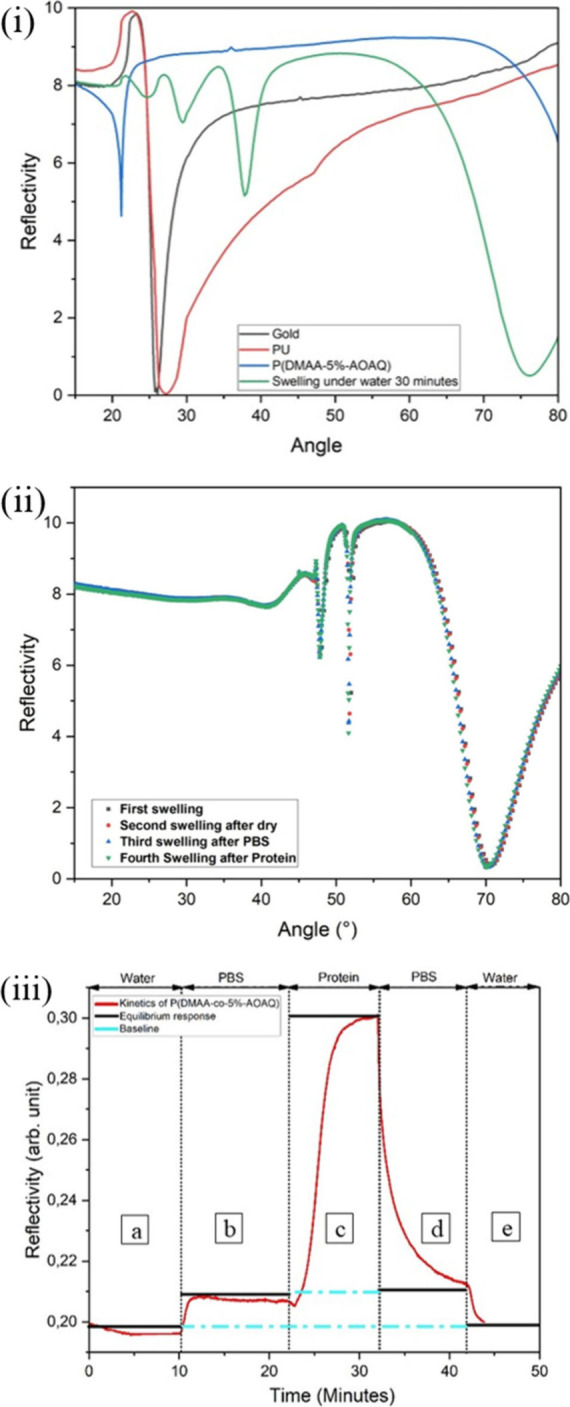
(i) Surface plasmon resonance (SPR) and Optical Waveguide (OWG) reflectivity curves (recorded under p-polarization) spectra were recorded to determine swelling, its stability in different media and protein adsorption of the hydrogel layers. (i) Reflectivity curves of the gold substrate (black curve) and a sample with 20 ± 1 nm polyurethane (PU) on top of the gold layer (red curve). Subsequently, a copolymer layer was applied via spin coating at 2000 rpm for 60 s, resulting in a thickness of 198 ± 5 nm, and treated under UV light at an intensity of 200 mW/cm^2^ for 3 min (blue curve). The swelling of the hydrogel layer was studied by immersing it in DI water for 30 min; the flow rate of the water was 40 μL/min (green curve). (ii) The swollen hydrogel thickness was evaluated in different media, including DI water (run 1), after drying and reswelling(run2), in PBS buffer (run2), and in a protein solution (run 4). In all cases, the thickness of the hydrogel in its swollen state remained consistent, confirming its stability under varying conditions. (iii) Reflectivity of the sample to determine protein adsorption onto the copolymer hydrogel layers at a fixed angle of R = 66°. To this P(DMAA-*co*-5%-(2-AOAQ)) was coated on the surface of a glass surface coated with layers consisting of 45 nm gold and 20 nm polyurethane. Measurements were performed at a flow rate 40 μL/min. Media: a) DI water b) Phosphate-Buffered Saline (PBS, pH 7.2–7.6) c) Human Blood Plasma Protein d) PBS buffer, e) DI water; Kretschmann geometry; λ = 632 nm, p-polarization. The swelling and protein repellency experiments were performed more than five times using different thickness measurements showing the reproducibility. These results were consistent in all experiments and were in close agreement with the results published in the literature.^44−48^

The swelling behavior of hydrogels in water was investigated using Surface Plasmon Resonance (SPR) spectroscopy and optical waveguide (OWG) spectroscopies. All reflectivity curves were recorded using p-polarized light. A gold-coated (∼43 ± 1 nm) substrate was first coated with a polyurethane PU layer (20 ± 1 nm) (4 mg/mL in THF) to ensure compatibility between the gold surface and the polymer layer. Subsequently, a copolymer solution (30 mg/mL in ethanol) was spin-coated onto the PU layer at 2000 rpm for 60 s, resulting in a dry copolymer layer thickness of 198 ± 5 nm, as measured (Figure 2(i)), indicated in blue). The layer was cross-linked under UV light (365 nm wavelength, 200 mW/cm^2^) for 3 min. For hydrophilic polymer coatings, all measurements were performed in a nitrogen atmosphere to exclude swelling effects from ambient humidity.

The SPR/OWG spectrum of the dry polymer layer (Figure 2(i), blue curve) revealed two distinct features. First, a sharp minimum at angles between 20° and 25°, resulting from the interference of the edge of total reflection and the first incoming waveguide mode. Second, the surface plasmon resonance (SPR) minimum would be observed at higher angles beyond 80°. The position of these features depends on the refractive index and thickness of the adsorbed polymer layer. Modeling the SPR/OWG reflectivity curves based on all refractive indices of the layers yields their thicknesses. To study swelling behavior, DI water was introduced into the system at a flow rate of 40 μL/min for 30 min. As shown in Figure 2(i) (green curve), the spectrum of a thick polymer film exhibited three waveguide modes. These modes allow simultaneous determination of both refractive index and layer thickness by iteratively modeling the lowest order mode (sensitive to the refractive index) and the highest order mode (sensitive to thickness). While minor deviations may arise for vertically inhomogeneous films, analyzing multiple modes resolves ambiguities present in thinner layers. SPR/OWG stimulation analysis revealed a significant increase in hydrogel thickness from 198 to 566 nm, corresponding to a swelling ratio of 2.5–4 (depending on the details of the layer composition and preparation), consistent with literature values.^44,48^

Further (Figure 2(ii)), the swelling behavior of the cross-linked copolymer layer in different media was investigated using Surface Plasmon Resonance (SPR). The copolymer was first swollen with water for 30 min, then dried using nitrogen, and subsequently reswollen. The same process was repeated for PBS buffer and a protein solution. SPR/OWG analysis revealed that, when the medium was changed no significant change in swelling of the hydrogel layer was observed, which is not surprising as the hydrogel layer does not contain any charges that might interact with the salt. This indicates and validates the structural stability and resistance of the copolymer layer to swelling-induced film degradation, highlighting its robustness in different environments.^44−48^

Later, the protein repellency of copolymer hydrogels was examined as it is a crucial factor in preventing cell adhesion and, subsequently, scar formation (Figure 2(iii)). The protein adsorption of the polymers was again studied using Surface Plasmon Resonance (SPR) spectroscopy.^45−48^ To this, the copolymer layer was exposed to deionized water, buffered solution (pH 7.2–7.6), and human blood protein, respectively, and the change in reflectivity was observed in the kinetics mode of the SPR experiment. To this first, a whole SPR spectrum was recorded, and then the angle was set to R = 66°, which is an angle just below the resonance angle of the surface plasmon. The reflectivity was recorded as a function of exposure time to the respective solution. As shown in Figure 2(iii), after changing the media from water to buffer, the reflectivity increases due to a slight change in the refractive index of the solution. Then, when protein solution was added during kinetics measurement, the reflectivity increased further, but upon washing, the reflectivity returned to the original baseline value, indicating that no protein had permanently adsorbed and the copolymer layer remained clean over the time frame of the experiment.^44,45,48^

**Figure 3:**
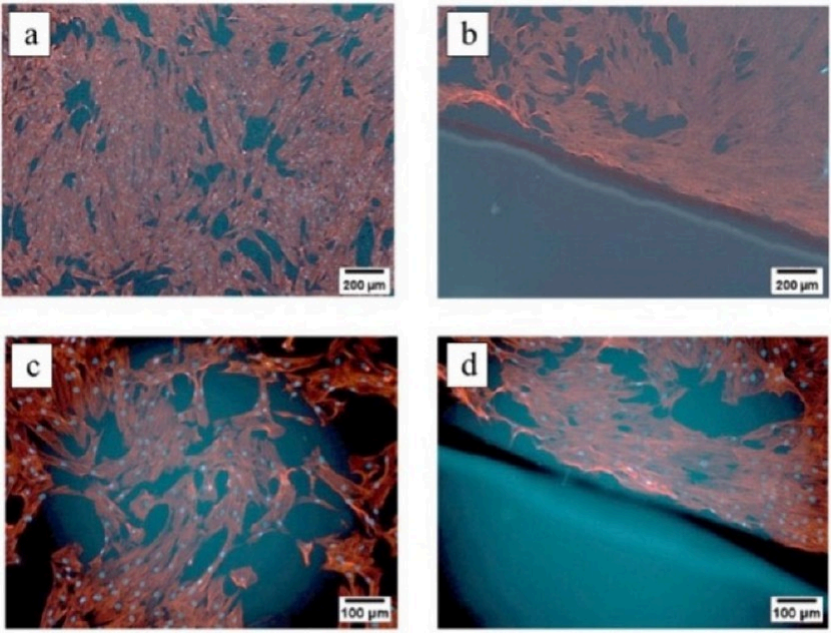
Fluorescence microscopic images of the HTF with the copolymer hydrogel patch on top, where a) Control/reference image of Human Tenon Fibroblast (HTF) cell culture on a coverslip, showing the F-actin cytoskeleton stained red and the nuclei in sky blue, which was not illuminated with UV light. The dark areas represent the empty spaces between cells. b) Image depicting the separation of the F-actin cytoskeleton and nuclei, with the hydrogel visible in the lower left corner, where the hydrogel was cross-linked with dose of 200 mW/cm^2^ for 3 min c) And d) Enhanced zoomed-in views of the same cell culture, with adjusted brightness for better clarity, providing a clearer view of the cell structure and interaction with the hydrogel, where the hydrogel is making a sharp edge with the cell nuclei and the F-actin cytoskeleton. This experiment also indicates that there are no cells on the top of the hydrogel, validating the protein/cell repellency of this hydrogel. The cell density was 10,000 cells per well in 24-well plates. The experiment was repeated three times to verify the results and ensure reproducibility.

In another set of experiments, coverslips were dip-coated so that half the surface was coated with the copolymer (20 mg/mL). The other half was left uncoated. The hydrogel on the half-coated coverslips was then cross-linked with UV light by irradiating with 200 mW/cm^2^ for 3 min and placed in the wells of a 6-well plate. After sterilization, Human Tenon Fibroblast (HTF) cells were placed in wells of a 6-well plate and incubated with the cell culture medium for 72 h. As shown in Figure 3, the experiment clearly demonstrated cell repellency against fibroblast cells. The cells avoided the coated area altogether, and no cells were observed on the top of the hydrogel surface. In contrast, they adhered very strongly to the noncoated areas, strongly indicating the effectiveness of the hydrogel in repelling fibroblasts.

### 3.3 Simulated Surgical Procedure on Porcine Sclera

During trabeculectomy, the eye is held in a clamp to prevent unnecessary movement. A scalpel is used to make an opening in the conjunctiva, (the filmy thin skin covering the surface of the eye), to access the sclera along with clearing the remaining adipose tissue, which is visible through this opening. A ’flap’ is then cut through part of the thickness of the tough white sclera. Under the flap, there is a small opening inside of the eye, toward the anterior chamber, where the eye fluid (aqueous humor) is located. Alternatively, this opening can be made at the edge of the iris to access the humor. This allows the fluid to flow under the flap and into a space created under the conjunctiva. Later, sutures are used to reattach the edges of the flap to the rest of the sclera so that the flap is loosely secured, creating a new drainage pathway for the aqueous humor to leave the eye, thereby reducing the pressure that causes glaucoma. The flowchart in the Supporting Information in Figure S10 explains the detailed step-by-step illustration of the hydrogel patch transfer.

**Figure 4:**
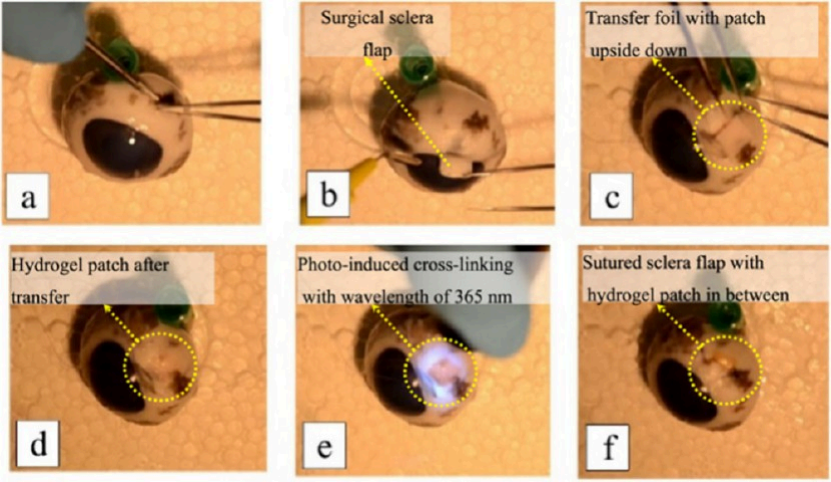
Patch application and surgery processes on porcine sclera following routine surgery protocols: (a) removal of connective tissue from the scleral surface; (b) creation of a thin scleral flap; and preparation to close the flap using surgical thread, securing the corners diagonally to the sides, (c) transferring and positioning the hydrogel patch on the sclera with a paper carrier; and gentle pressing of the patch to remove the carrier paper, (d) followed by removal of excess water and any necessary adjustments; (e) cross-linking of the patch with UV light illumination with dose of 200 mW/cm^2^ for 3 min; and (f) closing the flap with a minimum of three knots on each side. The experimental images appear yellow due to the use of a warm white light source. These simulated surgeries were repeated on more than ten porcine sclera samples, demonstrated reproducibility, successful transfer and consistent hydrogel attachment each time using the transfer foil.

This way, the entire surgical procedure was mimicked and performed on a porcine eye, as shown in Figure 4. The porcine eye was fixed to the polystyrene platform with a needle to prevent movement, and the conjunctiva and fatty tissue were completely removed from the ocular surface to allow access to the sclera of the eye (Figure 4a), as these could interfere with patch transfer and cross-linking. A surgical flap was then cut near the cornea to allow access to the interior of the eye. The eye was then preprepared for closure with the surgical suture to avoid further complications (Figure 4b). We then transferred the protein-repellent hydrogel patch together with the flexible transfer foil paper (Figure 4c), released the patch with a tiny drop of water as previously described (Figure 1b(iii)) and adjusted it with the paper and cotton swabs in the presence of a minimum of water (Figure 4d). The position of the transferred hydrogel patch was such that half of the patch was inside the flap. The rest was outside to maintain the flow and avoid scarring at this particular point. After successful transfer, the polymer in the hydrogel patch was cross-linked using UV light at a wavelength of 365 nm with the intensity of 200 mW/cm^2^ for 3 min (Figure 4e). The entire process, including patch transfer and cross-linking, takes approximately 4–5 min for this specific copolymer. The eye flap was closed back, and patch stability was checked with some routine tests such as water jet and swap scrubbing (Figure 4f). It should be noted that successful placement of these hydrogel patches can only be achieved by carefully reducing the presence of conjunctiva and adipose tissue on the sclera. If there are remnants of loosely attached adipose tissue on the sclera, the hydrogel will adhere to it, which can become a weak point in the attachment to the eye. Residual conjunctiva will create hydrogel coated bumps. It should also be noted that any significant excess of water should be removed with a cotton swab to prevent the hydrogel from curling. The water can be replaced after successful cross-linking of the copolymer. Failure to observe these conditions may result in uneven or damaged hydrogel patches, compromising the quality and efficacy of the fixation.

### 3.4 Cross-Sectioning of the Sclera

**Figure 5:**
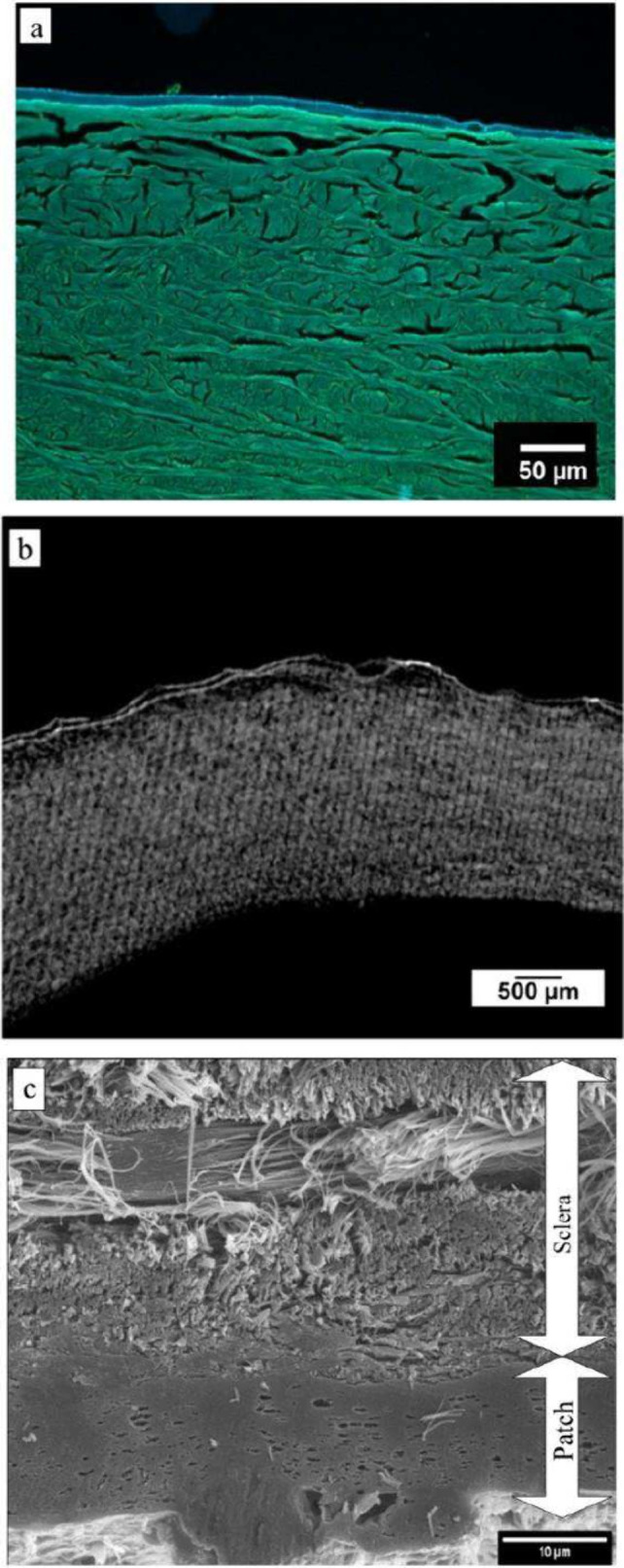
Cross-sectional images of the porcine sclera with the hydrogel patch on the top after cross-linking with the dose of 200 mW/cm^2^ for the duration of 3 min, and tissue fixation in all three cases (a) Fluorescence microscopy image of a scleral cross section showing the sclera (green) with the covalently attached hydrogel patch (blue) on top that was cross-linked, fixed and embedded in paraffin followed by sectioning. The layer transfer procedure was repeated more than ten times to validate reproducibility and consistency. All cross-sectional imaging consistently showed the patch firmly attached to the surface of the sclera, similar to the image presented in this figure. (b) 3D reconstruction of the images captured by Micro CT where the whole patch was examined for the interfacial attachment of the hydrogel patch, where all patches were cross-linked with 200 mW/cm^2^ for 3 min. Here, the top part with the double lines is the hydrogel patch and the part below is the scleral tissue, (c) SEM image displaying the thread-like collagen fiber structure of the sclera with the hydrogel patch (solid smooth layer below) closely attached, illustrating the detailed interface between the sclera and the patch along with the collagens. During Micro-CT and SEM imaging of 3–5 other patches with different cross-linking intensities for 3 min showed the same results as shown here.

The covalent attachment of the hydrogel patch to the sclera was successfully validated using fluorescence microscopy, with the formaldehyde fixed tissue embedded in paraffin. After fixation, the sclera was sectioned into thin slices of 5–10 μm thickness using a microtome, and these sections were mounted on glass slides for imaging. In Figure 5(a), the blue-colored line at the top represents the hydrogel patch, while the green-colored cracked surface corresponds to the sclera. The imaging confirmed the attachment between the hydrogel patch and the sclera remained largely intact.

However, as the hydrogel microtome section layers are very thin, and in many cases, partial removal or displacement was observed during cross-sectioning due to the shear forces exerted during the preparation of thin sections. To analyze the surface texture of the hydrogel, atomic force microscopy (AFM) imaging was performed at a specific point after transferring the hydrogel onto a polycarbonate glass slide. The corresponding images are provided in Figure S12 of the Supporting Information. Further, to better image the interface between the coating and the sclera, a nondestructive imaging technique, micro-CT, was used as it can provide detailed 3D scans. In the micro-CT study of the scleral samples (Figure 5b), after successful transfer and cross-linking, the sclera and hydrogel patch were fixed using a critical point dryer (CPD). The dried sclera exhibited a lightweight, Styrofoam-like appearance, with an intact tissue surface and an undamaged hydrogel patch. The sclera was then sectioned into smaller, subcuboidal pieces to examine the interaction and porosity at the interface. Micro-CT imaging (Figure 5b), performed across multiple locations of the same scleral sample, showed a homogeneous sclera coating and revealed no air bubbles or cavities at the interfacial junction. This finding corroborates the fluorescence microscopy results, confirming uniform attachment across the surface after cross-linking. The absence of interfacial defects further demonstrates the bond’s integrity between the hydrogel and the sclera and the consistency of the transfer process.

For higher-resolution imaging of the interface between the sclera and the hydrogel, scanning electron microscopy (SEM) was used to capture cross-sectional images. As shown in Figure 5c, the top portion of the image reveals the fibrous structure of the scleral collagen, characterized by a dense and intertwined network, and below is the solid, smooth layer representing the hydrogel patch. The SEM images in Figure 5c clearly demonstrate the penetration and interlocking between the fibrous scleral collagen and the hydrogel at the interface. This close interface suggests that the hydrogel film is tightly adhered to the collagen fibers without any air gaps or nonuniformity in the attachment. Additionally, the image highlights the presence of only a few tiny, nonconnected pores within the interface, each with an approximate diameter of less than one micron.

As the pore density is very small, they do not disrupt the overall adhesion, allowing the hydrogel to fill even the smallest cavities on the scleral collagen fibrous surface, which in turn ensures a secure and strong attachment.

### 3.5 Effect of UV Irradiation

Choosing the correct irradiation dose is crucial for the success of the process. Prolonged exposure to short-wavelength UV light can damage both copolymers and healthy tissues. High-energy UV light can generate reactive oxygen species (ROS, which can cause cellular damage). This can induce DNA damage, protein oxidation, and lipid peroxidation, which can compromise cell viability and function, leading to trauma or inflammation. On the other side, if the UV irradiation is insufficient, the hydrogel patch may cross-link but fail to attach properly to the sclera.^37,48^

To investigate the effects of UV illumination, the presence of H2A, a marker for DNA repair, was monitored. During DNA damage, modification of Histone 2A, such as phosphorylation, notably the H2AX variant, are the earliest cellular responses. These modifications signal the recruitment of DNA repair machinery to the site of damage, facilitating the repair of broken DNA strands. Thus, coverslips coated with Human Tenon Fibroblast (HTF) cells were taken as a model system and illuminated with UV light of a wavelength of 365 nm at different doses but a fixed time of 3 min.

**Figure 6:**
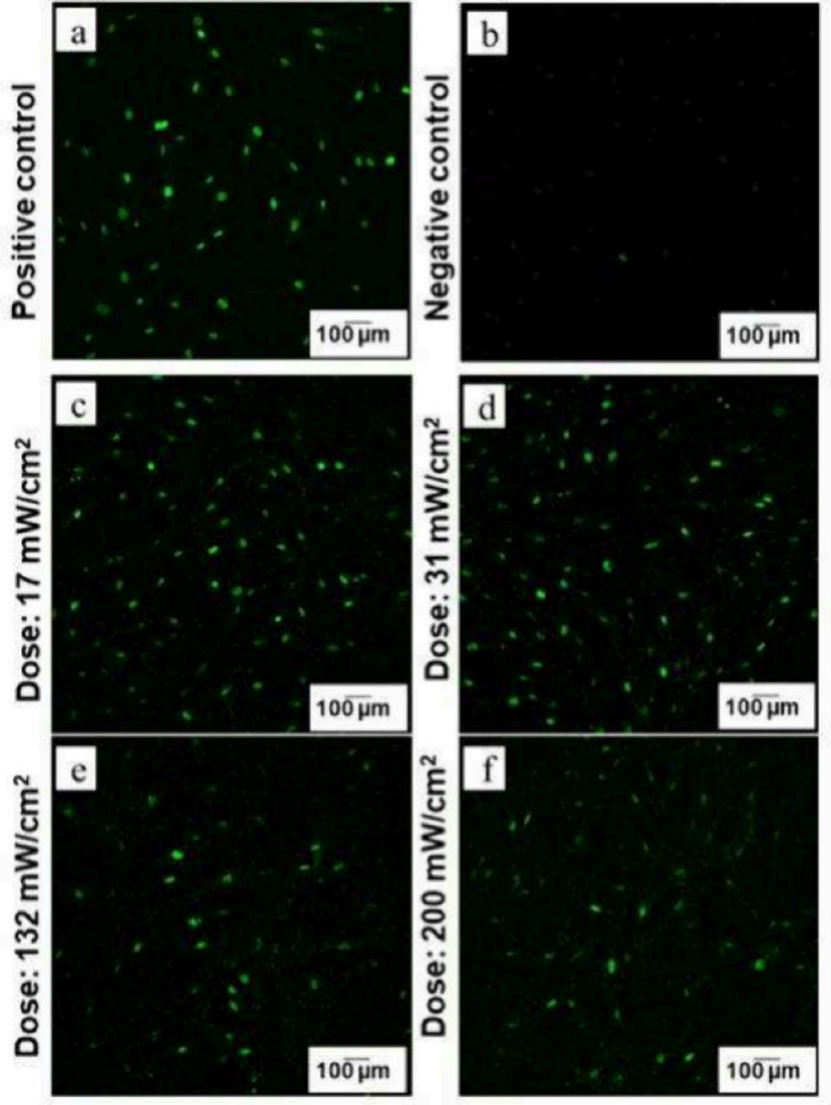
Fluorescence Imaging of H2A Release in Cell Nuclei Under Varying UV Illumination Conditions; dye = secondary antibody goat antirabbit, Alexa Fluor 488 nm (a) positive control, where cells were illuminated with UV light at an intensity of 200 mW/cm^2^ for 10 min; increased brightness in the fluorescence image confirms the successful detection of H2A release, (b) negative control shows cells that received no UV treatment. As expected, no significant green fluorescence is observed, confirming the absence of H2A release. (a and b) establishes a baseline for comparison. (c), (d), (e), and (f) images depict H2A release in cells exposed to 365 nm UV light for 3 min at varying intensities. The varying brightness levels across these images demonstrate the dose-dependent effect of UV illumination on H2A release. The cell density was 10,000 cells per well in a 24-well plate. The cell staining experiment using H2A, DAPI and Phalloidin stains as an indicator of UV illumination was performed in triplicate across in more than five experimental runs, with consistent success in all attempts.

This way, we vary the UV dose but keep the time frame of the experiment the same, as this is important for any planned surgery procedure later in vivo. The samples were stained by adding fluorescently labeled antibodies against H2A (labeled with secondary antibody goat antirabbit, Alexa Fluor 488 nm) along with DAPI and Phalloidin. Here in Figure 6, the presence of H2A is indicated by a green fluorescence, in which a brighter spot indicates the more extensive release of H2A in the nuclei. In contrast, the lighter-colored or dotted spots indicate a lower release of H2A. The celĺs nuclei can be observed in blue color because of DAPI and the F-actin cytoskeleton with red stain because of the Phalloidin fluorescence indicator in the HTF cells. The fluorescence images corresponding to all the stains can be found in the Supporting Information (Figure S5).

In Figure 6a, the positive control is shown, which was illuminated with 200 mW/cm^2^ for 10 min to observe UV-induced cell damage and, accordingly, the strong release of the H2A. The cell culture showed many green spots in the fluorescence images, indicating the intense release of H2A. The negative control was not illuminated; thus, no fluorescence signals were observed, indicating no release of the H2A (Figure 6b). In Figure 6(c-f), it is observed that there was a significant presence of H2A at several positions independent of the irradiation dose of the UV light. This indicates that even at the lower dose (Figure 6c) and for a shorter duration, some cell nuclei will experience some, albeit much smaller, changes compared to the positive control. Thus, it can be concluded that the DNA or cell repair mechanism is released when the tissue is illuminated with UV light, independent of the dose and the time duration.

**Figure 7:**
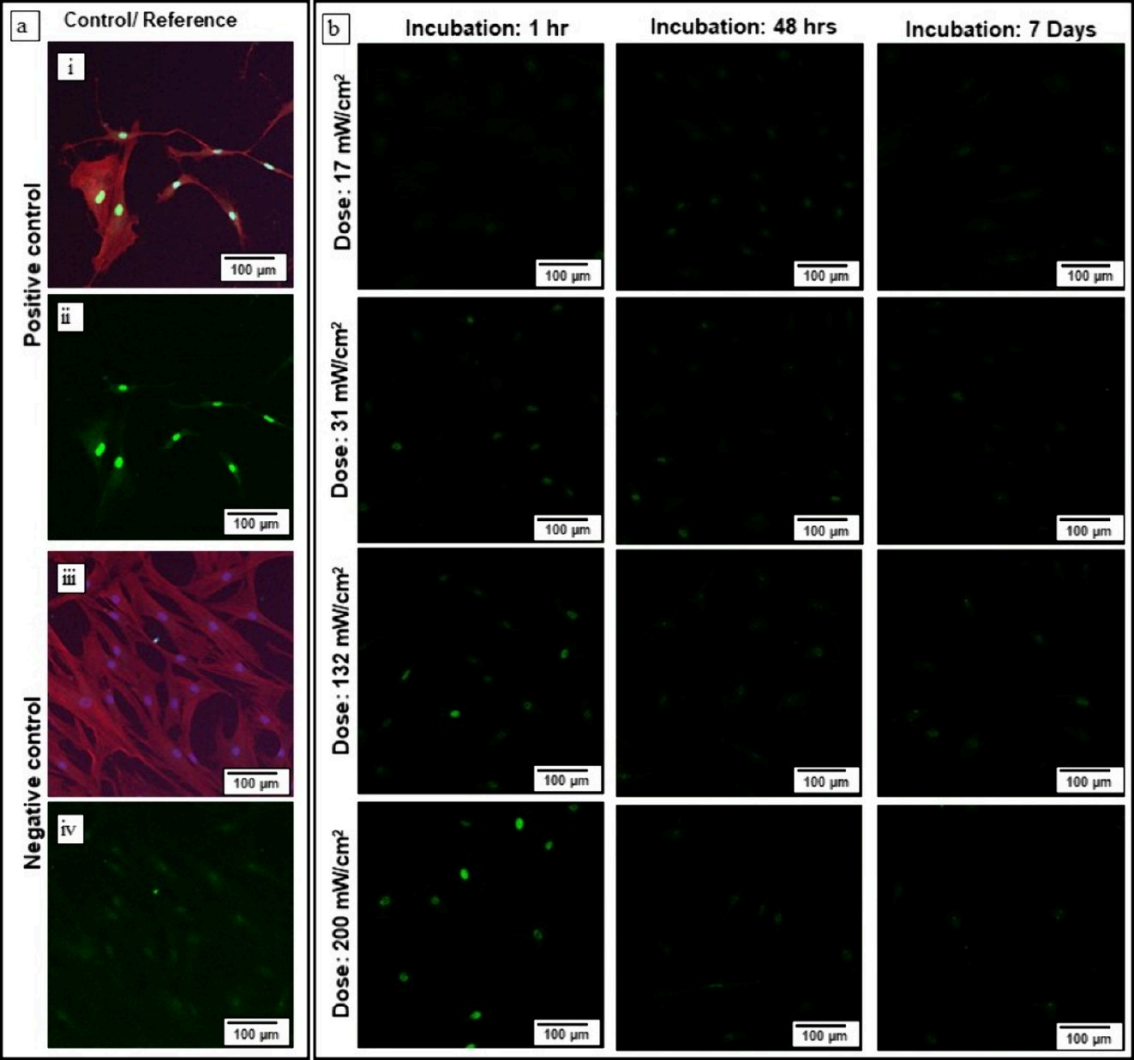
Analysis of H2A contents of cells as a function of incubation time and UV illumination dose. In all images, the red color represents the F-actin cytoskeleton stained with Phalloidin, blue color represents DAPI. The bright green fluorescence marks the presence of H2A, with its intensity serving as a quantitative indicator of H2A release. (a) control/reference for H2A presence including a positive control (a(i, (ii)), where cells were subjected to a high UV dose of 200 mW/cm^2^ for 10 min and a negative control with no UV treatment (a (iii, (iv)). (a (i, (iii)) is the merged image of DAPI, Phalloidin and Alexa Fluor 488 nm. (b): experimental results; columns corresponding to different incubation periods and rows representing various UV illumination doses applied to the cells for 3 min. The green fluorescence in each image reflects the amount of H2A released under each condition. The results indicate that while cells exhibit an initial response to UV-induced environmental changes, characterized by H2A release, they show significant decrease in release H2A in the HTF cells nuclei from 1 to 48 h and later a complete recovery within a 7-day incubation period. The cell density was 10,000 cells per well in a 24-well plate. The cell staining experiment utilizing H2A, DAPI, and Phalloidin stains as indicators of UV illumination was conducted in triplicate across multiple experimental runs, achieving consistent success in all cases.

However, not only the direct response to the irradiation, but also the recovery of the cell’s nuclei and the subsequent decrease in the H2A contents in the system is essential for the success of the process. To elucidate this, we next study the recovery of the cells during incubation, where any recovery is expressed by a decrease in the H2A contents in the system. When the cell recovers, the H2A contents becomes reduced, and the green fluorescence signals in the applied test disappear. To this, the HTF cell cultures were first exposed to UV irradiation with different intensities with a constant illumination time of 3 min. Then, the cell cultures were incubated in cell culture medium for varying times, and the samples were imaged again for the H2A present in the system. As shown in Figure 7a (i, (ii), in the positive control, the fluorescence signal intensity due to H2A recruitment remained unchanged, and identical fluorescence signals were observed even after prolonged incubation. In the negative control (Figure 7a (iii, (iv)), also no change was observed, and the fluorescence remained low. In Figure 7a (i and (iii), the images of the merged secondary antibody goat anti rabbit, Alexa Fluor 488 nm, and DAPI signals were overlaid with those of Phalloidin signals where Phalloidin stained the F-actin cytoskeleton of the cells (indicated red). In Figure 7b, the incubation was performed for different durations, such as 1 h, 48 h, and 7 days. Here, it is observed that the signal intensities of the fluorescence signals are becoming strongly reduced with time, indicating the H2A variant concentration decreases drastically in every cell independent of the irradiation dose. The H2A level decreases strongly to almost zero after 48 h incubation, leading to complete vanishing after 7 days of incubation. This might indicate that the cells have entirely recovered. The corresponding fluorescence images with different staining like Phalloidin, Alexa Fluor, and DAPI of the cells are shown in the Supporting Information along with the merged images of all the stains, for a detailed understanding. The figures corresponding to different time points are as follows: 24 h (Figure S6), 48 h (Figure S7), and 7 days (Figure S8).

**Figure 8:**
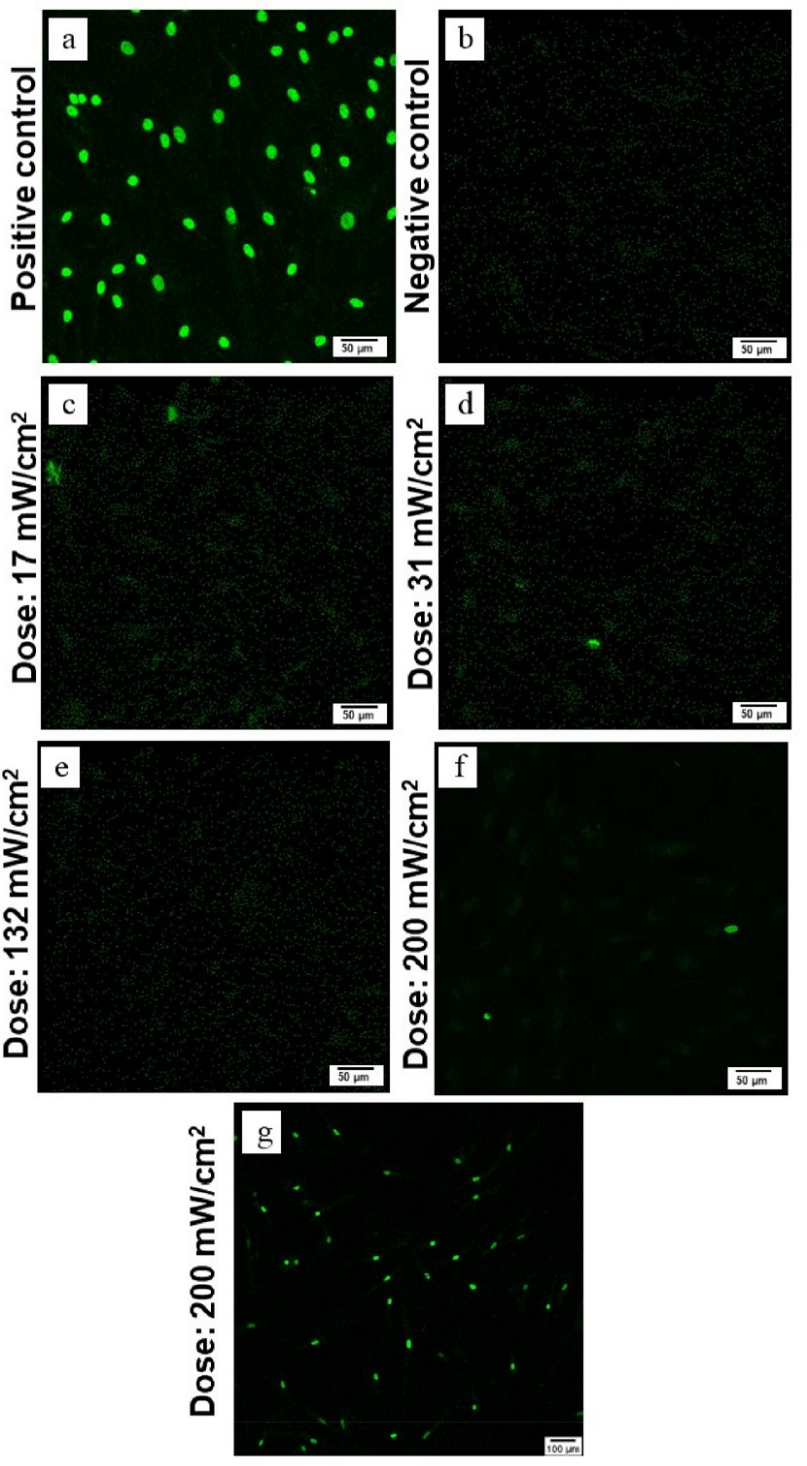
Fluorescence imaging of cells after UV illumination with various doses and addition of DAPI and fluorescein 12-dUTP as fluorescence indicators in an apoptosis test. Green spots are caused by the fluorescence indicator Fluorescein 12-dUTP, which indicates apoptotic cells. (a) After treating the cells with DNase, an enzyme degrading DNA to fragmentation, positive control indicates apoptotic green spotted cells (b) Negative control with the same process, but no UV irradiation or DNase. (c), (d), (e), and (f): These panels display the results of cells exposed to UV light at varying energy doses for 3 min. The absence of green fluorescence in each image indicates no presence of apoptotic cells. (g) It shows that cells exposed to UV light for an extended duration of 10 min with the dose of 200 mW/cm^2^, indicating green fluorescence, provide insight into the cumulative effect of prolonged UV exposure on inducing apoptosis in HTF cells. The cell density was 10,000 cells per well in a 24-well plate. The cell staining experiments, using 12-dUTP, and DAPI stains as markers of UV-induced damage, were performed three times during multiple experimental runs (more than five) and consistently yielded successful results. Strict adherence to the established protocol ensured reliable and error-free outcomes.

To exclude that cell death led to a loss of the fluorescence signals in the H2A study, we next studied the apoptosis of the HTF cells following UV illumination at a wavelength of 365 nm. This was carried out through the fluorometric TUNEL staining test on the fibroblast cells.^49^ To this, the HTF cells were prepared on coverslips in wells of 6-well cell cultured plate and irradiated at different intensities. In Figure 8, the control shows apoptotic cells when the HTF cell culture, stained with DNA strand breaks fluorescein 12-dUTP, was exposed to Deoxyribonuclease (DNase), which is an enzyme for degrading DNA to fragmentation. The bright green color indicates the presence of a large number of apoptotic cells because of fluorescent indicator **FITC** (Fluorescein 12-dUTP) (Figure 8a). In contrast, the negative control (Figure 8b), left without irradiation or DNase, and showed no signs of apoptosis. As shown in Figure 8c-f, after irradiation at different doses for the duration of 3 min and incubation, there was no indication of apoptosis.

If we now vary the illumination time but use the maximum intensity of 200 mW/cm^2^ while keeping all other conditions constant, it was observed that starting from 10 min of irradiation, significant apoptotic activity starts becoming evident in the cell culture at multiple sites (see Figure 8(g)).

Thus, all these findings highlight that H2A release was successfully observed across nearly all cells, with an impressive 99% cell recovery across the coverslips. A minor fraction (<1%) of apoptotic cells was noted at isolated locations, likely due to cell handling variations or the presence of foreign particles. Notably, when specific conditions were applied such as light intensities between 17 to 31 mW/cm^2^, the release of H2A was reduced compared to higher intensities of 132 and 200 mW/cm^2^, indicating a controlled and manageable response under optimized settings. Hence, even with a wide set of conditions, where the cells experience no permanent damage, it is essential to carefully balance UV intensity and exposure duration to avoid detrimental effects on cell viability and the mechanical stability of the hydrogel patch. Too high UV doses make the gel rigid and brittle because it might lead to chain breakage.^48^ Therefore, in all following experiments, an intensity of 17 mW/cm^2^ was used as this allowed complete cross-linking and caused only minimal cell damage while keeping the irradiation time of 3 min. The fluorescence images of HTF cells exposed to the respective doses obtained by DAPI, Fluorescein 12-dUTP stains, and merged images of all staining are provided in the Supporting Information for the respective dosages in Figure S9 and S11.

### 3.6 Stability

During glaucoma surgery, on the one hand, mechanical stability is a crucial parameter, and on the other hand, stability against medication is also essential. For the prior, it should be noted while the hydrogels have Young’s moduli only on the order of a few 10 kPa, during the surgical procedure they are supported by a foil backing and are only released on the desired location of the eye. After deposition they are extremely slippery and show strongly lubricious behavior.^33,34^ In subsequent studies, we will add more measurements on the tribology and wear behavior of such systems.

For the latter aspect, i.e., stability against medication, Mitomycin C or steroids are used for the latter to treat the sclera and surrounding connective tissue. To address this stability, we investigated the effects of common postoperative medications like mitomycin-C, regular eye drops, and steroid therapy with Fortecortin (an anti-inflammatory medication) on the eye covered with the hydrogel patch.

**Figure 9:**
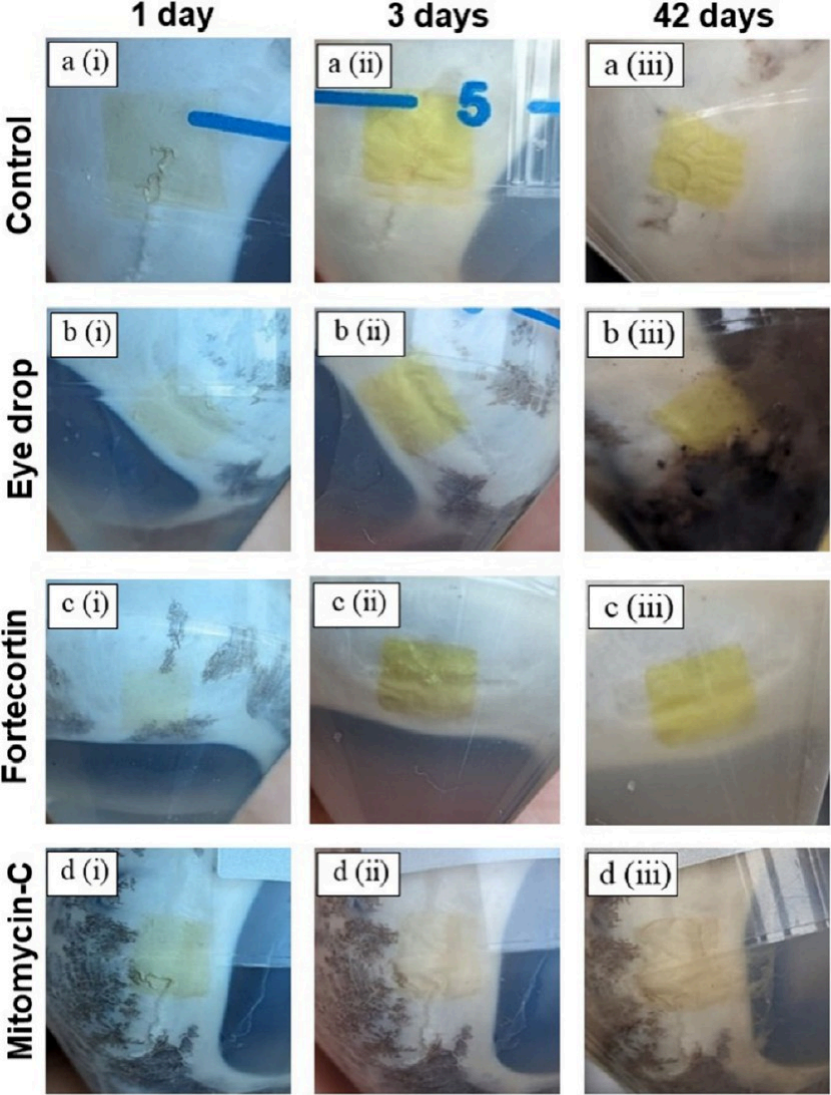
Stability of Cross-linked Hydrogel Patches on Porcine Eyes in Various Environments. cross-linked hydrogel patches are placed in 50 mL centrifugation tubes, each containing 20 μL of different drug solutions in 20 mL PBS (pH 7.2–7.6) to evaluate the stability of the hydrogel patches under the influence of drugs used in trabeculectomy surgery (a (i, ii, (iii)) represent the control group which, serves as the reference. The eyes are immersed in a phosphate-buffered saline (PBS) solution without drug interference. (b (i, ii, (iii)): porcine eyes in PBS with the addition of standard eye drops, which are primarily saline-based, demonstrating the hydrogel patch’s structural integrity in this solution, indicating no adverse effects from the saline. (c (i, ii, (iii)): porcine eyes in PBS with Fortecortin, an anti-inflammatory drug, indicating that the presence of Fortecortin does not compromise the hydrogel’s functionality. (d (i, ii, (iii)): Porcine eyes in PBS with Mitomycin-C, a highly toxic drug that prevents scarring during surgery. In this proof of principle study, one sample per drug was used.

The treated eyes, together with the patches, were immersed in 20 mL PBS (pH 7.2–7.6) containing 20 μL of the selective drugs and subjected to an ultrasonic bath twice daily to simulate blinking and eye rubbing. The patch was found to sit stably on the sclera with no visual deformation of the patch or partial leakage (Figure 9). The observations and images recorded over a maximum period of 42 days showed that the patch remained intact and adhered to the sclera. After this, the porcine eye began to degrade. However, even then, when small pieces of sclera tissue were taken, which do not degrade in a sterile, closed environment and are occasionally ultrasonicated, the experiments showed that the patch was still adhering to the sclera for more than 1.5 years (not shown). This demonstrates the excellent adhesion and resilience of the hydrogel patch and its compatibility with postoperative treatments.

## 4 Conclusion

Complex biomaterial surfaces like those present in the eye can be efficiently modified by depositing a protein-repellent hydrogel patch using gel transfer followed by attachment to the ocular surface by C, H insertion cross-linking. The protein-repellency leads to a strongly reduced cell adhesion which in turn leads to a reduction of the adhesion on undesired cells, which can cause scar formation. A paper-based flexible foil, which is on one side hydrophobic and on the other side hydrophilic (“tattoo paper”), has proven to be an excellent temporary backing for the thin layer to be placed on the surface. After placement on the eye the addition of just a few drops of water is sufficient for a fast and efficient release (gel transfer). The photoreaction, which leads to a covalent linking of the forming hydrogel patch to the ocular surface, causes excellent adhesion between the gel and the eye surface. This is even in wet environments the case such as the permanently moist surface of functioning ocular tissue. During in vitro studies and following simulated operational processes, the hydrogel patch can be easily cut to the desired size, applied to the tissue, and released from the paper backing and photo cross-linked within just a few minutes. Currently, the whole process takes about 4 min, but a shortening to 2 min is feasible. Remarkably, the hydrogel patch has shown the ability to adhere to porcine sclera for more than a year without any damage or detachment on the sclera surface. While initially, the cells of the ocular tissue show some response to the UV radiation, as long as the irradiation dose is kept within limits, they quickly recover and show no further signs of damage. All in all, this approach, where a strong reduction of cell adhesion is achieved, promises to avoid scar formation after trabeculectomy and might turn into a long-term solution for managing glaucoma scarring.

The straightforward production and application processes, as well as the short handling times, make this technique easily used in any laboratory or surgery room. Consequently, this method of hydrogel patch transfer has been validated as a simple and versatile approach for the targeted modification of cell adhesion to tissue. It holds significant potential for advancing tissue engineering as it allows very strong control of the positioning of the cells on any biomaterial surfaces. We now focus on in vivo experiments to explore its capabilities further.

## Supporting Information Available

Polymer characterization and the detailed experimental results, including the different intensity cross-linking paraffin sectioning, the cell culture, and the indication of recovery or apoptosis of the cells (PDF)
Micro-CT reconstruction of sclera with patch (MP4)

## Abbreviations

DMAA: N,N-Dimethyl acrylamide
AOAQ: 2-Acryloxyanthraquinone
CHic: Carbon–hydrogen insertion cross-linking
UV: Ultraviolet
min: minutes
NMR: Nuclear magnetic resonance
SPR: Surface plasmon resonance
SEM: Scanning electron microscopy
hrs: hours
eq: equivalent
mW/cm^2^: milliwatts per centimeter square
J/cm^2^: joules per centimeter squared
g/mol: grams per mol
mg/mL: milligram per milliliter
μL: microliters
DNase: Deoxyribonuclease
