# Supplementary material for: Attachment of Hydrogel Patches to Eye Tissue through Gel Transfer using Flexible Foils
Source: ACS Appl Mater Interfaces. 2025 Jan 28;17(6):8849–61. doi: 10.1021/acsami.4c15089 (PMC11826510; doi:10.1021/acsami.4c15089)
Supplement: Supplementary file 1 — am4c15089_si_001.pdf [file am4c15089_si_001.pdf]

Supporting Information (SI)

Attachment of Hydrogel Patches to Eye Tissue through Gel Transfer using Flexible Foils.

Shubham Tiwari[1], Luisa Goldmann[3], Jan Lübke[3], Oswald Prucker[1,2], Gottfried Martin[3], Günther Schlunck[3] and Jürgen Rühe[1,2]*

[1] Department of Microsystems Engineering (IMTEK), Laboratory for Chemistry & Physics of Interfaces (CPI), Albert-Ludwigs-Universität Freiburg, Georges-Köhler-Allee 103, 79110 Freiburg, Germany

[2] Cluster of Excellence livMatS @ FIT–Freiburg Center of Interactive Materials and Bioinspired Technologies Albert-Ludwigs-Universität Freiburg, Georges-Köhler-Allee 105, 79110 Freiburg, Germany

[3] Eye Center, Medical Center – University of Freiburg, Faculty of Medicine, University of Freiburg, Killianstraße 5, 79106 Freiburg, Germany.

1) Monomer synthesis of 2-Acryloxyanthraquinone (AOAQ):

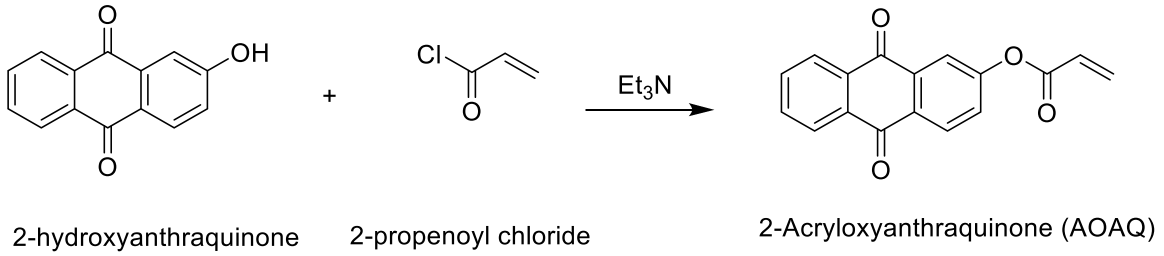

Figure S1. *Reaction diagram of the representation of monomer synthesis 2-Acryloxyanthraquinone.*

4 g of 2-hydroxyanthraquinone (17.8 mmol, 1 equivalent, 224.21 g/mol) was dissolved in 350 ml dichloromethane under nitrogen. The mixture was cooled with an ice bath, and 4.8 ml of triethylamine (35.7 mmol, 2 equivalent, 101 g/mol) was added. Then, 4.3 ml of acryloyl chloride (53.5 mmol, 3 equivalent, 90.51 g/mol) in 30 ml dichloromethane was added dropwise. The mixture was left until reaching room temperature and it was stirred overnight. The organic phase was washed two times with 0.1 M HCl and then two times with distilled water. Next, it was washed two times with saturated NaHCO3 solution and then two times with distilled water. The remaining organic phase was dried over sodium sulphate (anhydrous), and ¾ of its volume was removed under reduced pressure. After that, the product solution was added dropwise into fivefold cold methanol. The flask was stored in the freezer for crystallization. The product was collected, washed with cold methanol, and dried under vacuum overnight**.**

2) Copolymer synthesis of P(DMAA-co-5%-(2-AOAQ)):

In a steering Schlenk tube, the raw product solution of 2- Acryloxyanthraquinone (AOAQ) (556 mg, 2 mmol, 1 eq.) was dissolved together with *N, N*-Dimethylacrylamide (DMAA, 3.767 mg, 38 mmol, 19 eq.) in 40 mL DMF. To the solution was then added 2,2-azobis (4-methoxy-2,4-dimethylvaleronitril, AMDVN, 1.8 mg, 0.058 mmol). The reaction solution was degassed by six times freezing, vacuum pulling, and thawing ("*freeze-pump-thaw*") and polymerized under vacuum at 30°C 72 hrs. Afterward, the resulting polymer solution was ventilated by dropping in cold in 600 ml of diethyl ether with strong stirring. The lump was decanted, dissolved in little chloroform, and further felled twice in cold diethyl ether. The resulting polymer was extracted and dried in a high vacuum.

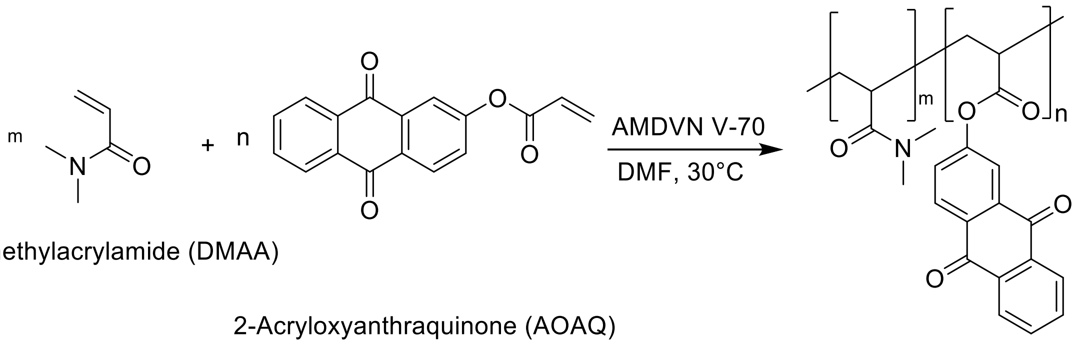

N,N-Dimethylacrylamide (DMAA)

2-Acryloxyanthraquinone (AOAQ)

P(DMAA-co-5%-AOAQ)

Figure S2. *Reaction diagram of the representation of copolymerisation of 2-Acryloxyanthraquinone (AOAQ) with N,N dimethylacrylamide (DMAA)*

3) Material Characterization:

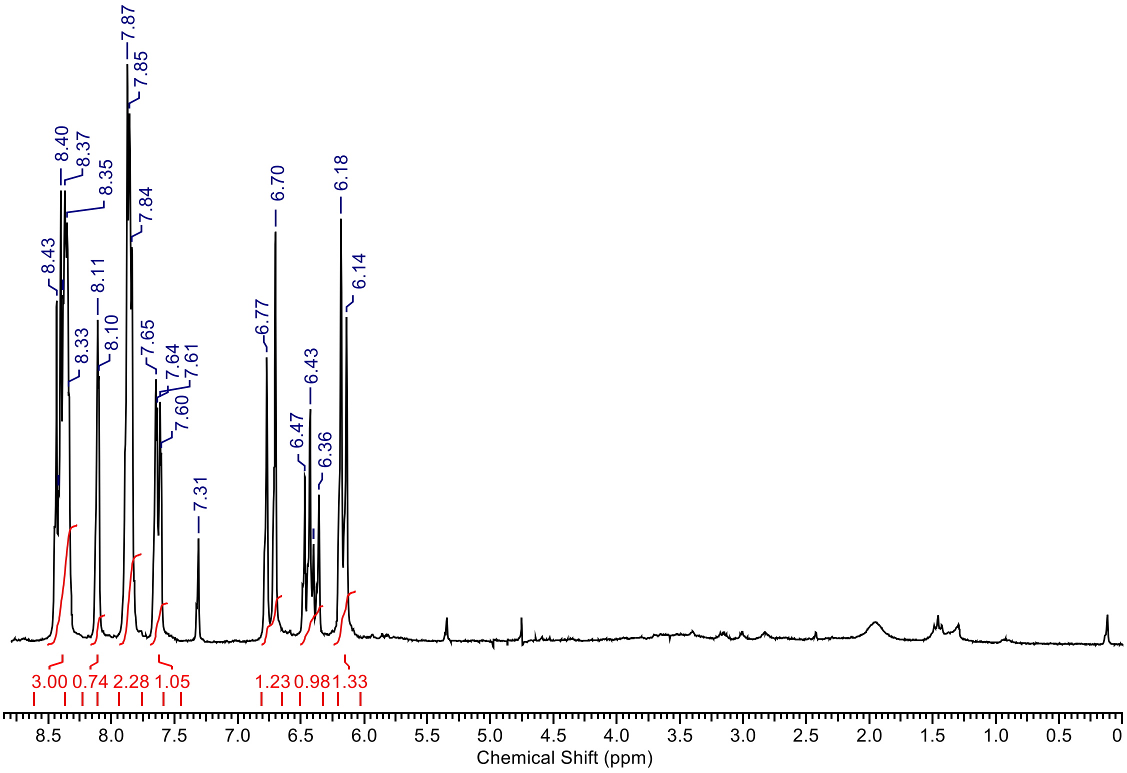

Figure S3*. ^{1}H NMR spectra of the monomer 2-Acyryloxyanthraquinone (2-AOAQ)*

1H-NMR (250 MHz, CDCl3): δ (ppm) = 8.43 – 8.33 (d, ^{3}J = 8.5 Hz, 1H, CH_{ar}), (m, 2H,CH_{ar}), 8.10-8.11 (d, ^{4}J = 2.4 Hz, 1H, CH_{ar}), 7.84 – 7.87 (m, 2H, CH_{ar}), 7.60 - 7.65 (dd, ^{3}J = 8.5 Hz, ^{4}J = 2.4 Hz,1H, CH_{ar}), 6.70 - 6.77 (dd, ^{3}J = 17.2 Hz, ^{2}J = 1.2 Hz, 1H, CH=CHH), 6.36 - 6.47 ((dd, ^{3}J = 17.2 Hz, ^{3}J = 10.4Hz, 1H, CH=CH2), 6.14 – 6.18 (dd, ^{3}J = 10.4 Hz, ^{2}J = 1.2 Hz, 1H, CH=CHH).

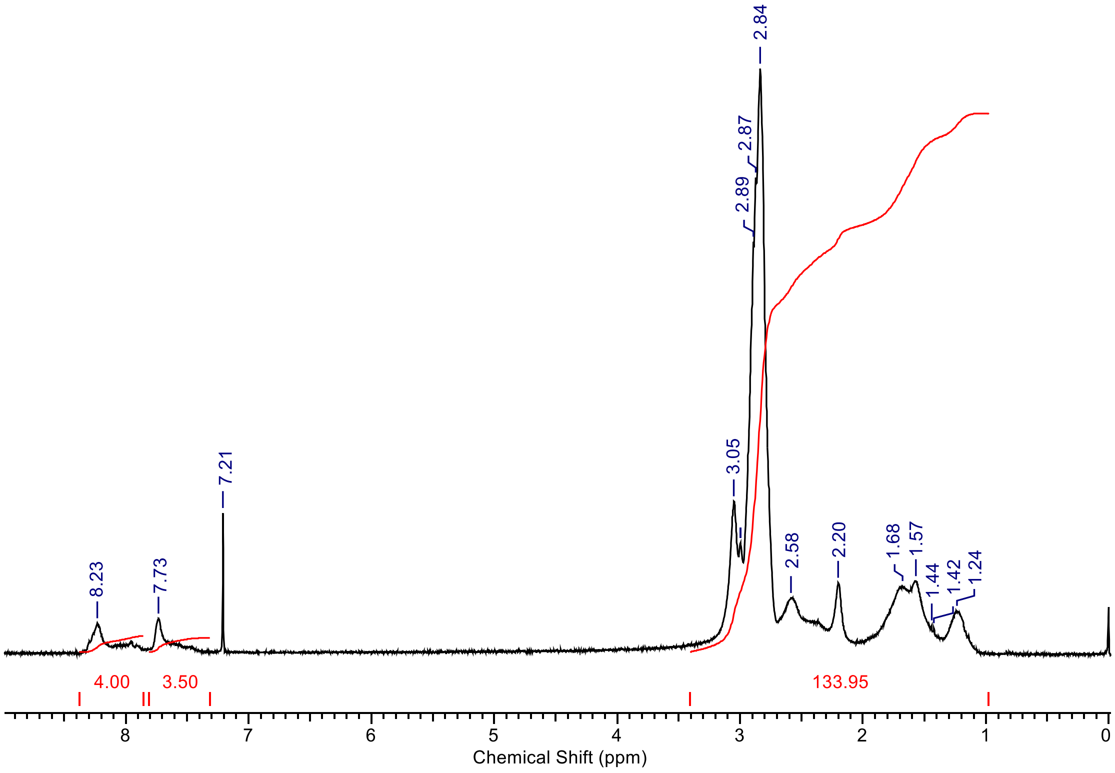

Figure S4. *^{1}H NMR spectra of the copolymer of N,N Dimethylacrylamide and 5% of 2-Acryloxyanthraquinone P(DMAA-co-5%-(2-AOAQ)).*

^{1}H-NMR (250 MHz, CDCl3): δ (ppm) = 1.1-1.8 (C*H2*-CH) 2.0-2.8 (N-(C*H3*)2), 2.9-3.3 (C*H*-CH2) 7.3-8.4 (CAr-*H*)

GPC (DMF): Mn = 7.56x10^4 – 1.05x10^5 g/mol, Mw = 2.83x10^5 3.15x10^{5}g/mol, PDI = 3.74-4.0.

4) Effect of UV irradiation with H2A recovery or apoptosis:

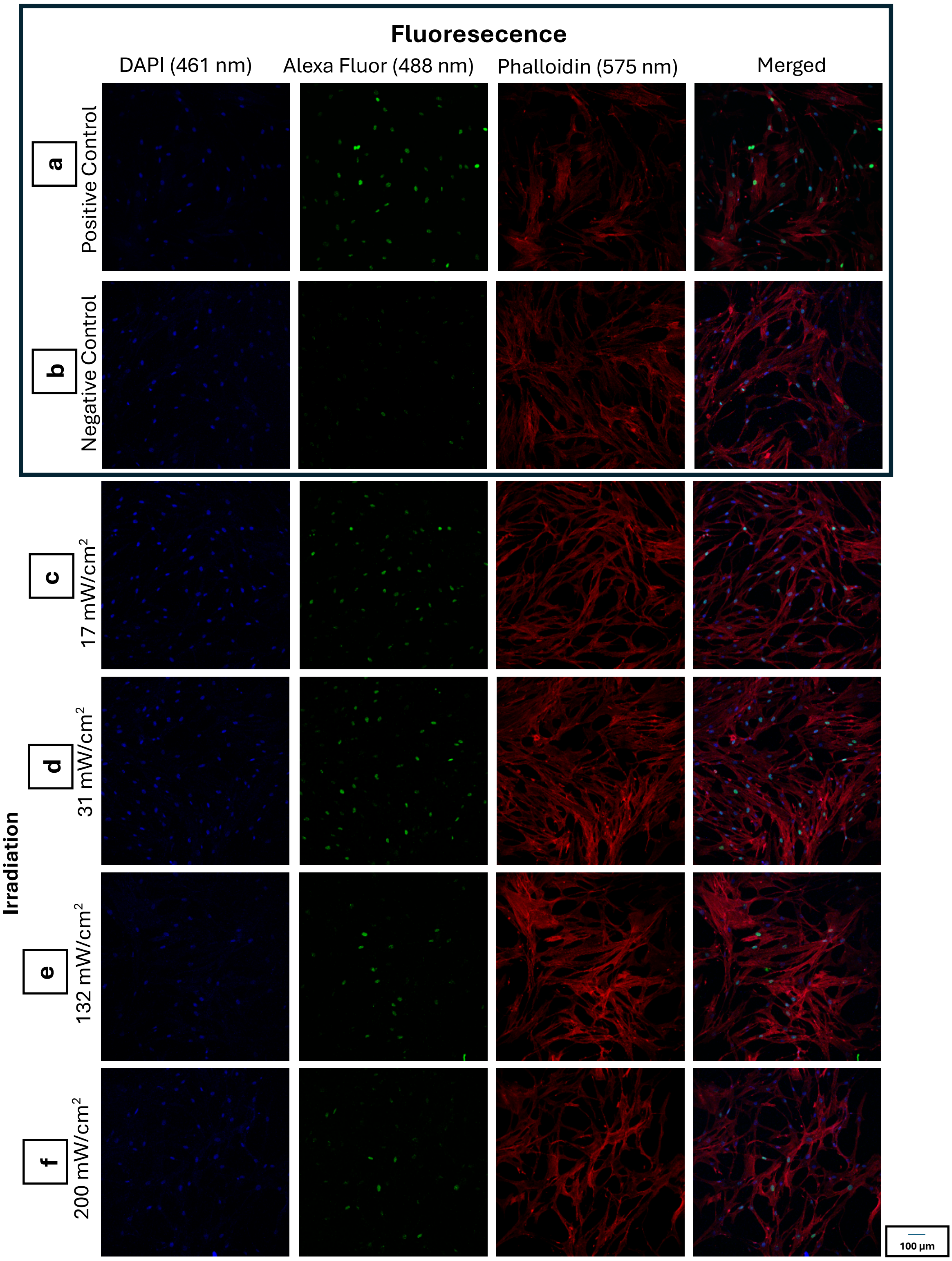

Figure S5. *Fluorescence Imaging of H2A Release in Cell´s nuclei Under Varying UV Illumination Conditions; dye = DAPI (nuclei indicator in blue), secondary antibody goat anti-rabbit, Alexa Fluor 488nm (H2A stained green) and Phalloidin (F actin cytoskeleton stained red). In the figure´s row, (a) positive control, where cells were illuminated with UV light at an intensity of 200 mW/cm² for 10 min; increased brightness in the green fluorescence image confirms the successful detection of H2A release, (b) negative control shows the cells that received no UV treatment. As expected, no significant green fluorescence is observed, confirming the absence of H2A release. (a & b) establishes a baseline for comparison. (c), (d), (e), and (f) images depict H2A release in cells exposed to 365 nm UV light for 3 minutes at varying intensity doses. The varying brightness levels across these images demonstrate the dose-dependent effect of UV illumination on H2A release. The cell density was 10,000 cells per well in a 24-well plate. The cell staining experiment using H2A, DAPI and Phalloidin stains as an indicator of UV illumination was performed in triplicate across in more than five experimental runs, with consistent success.*

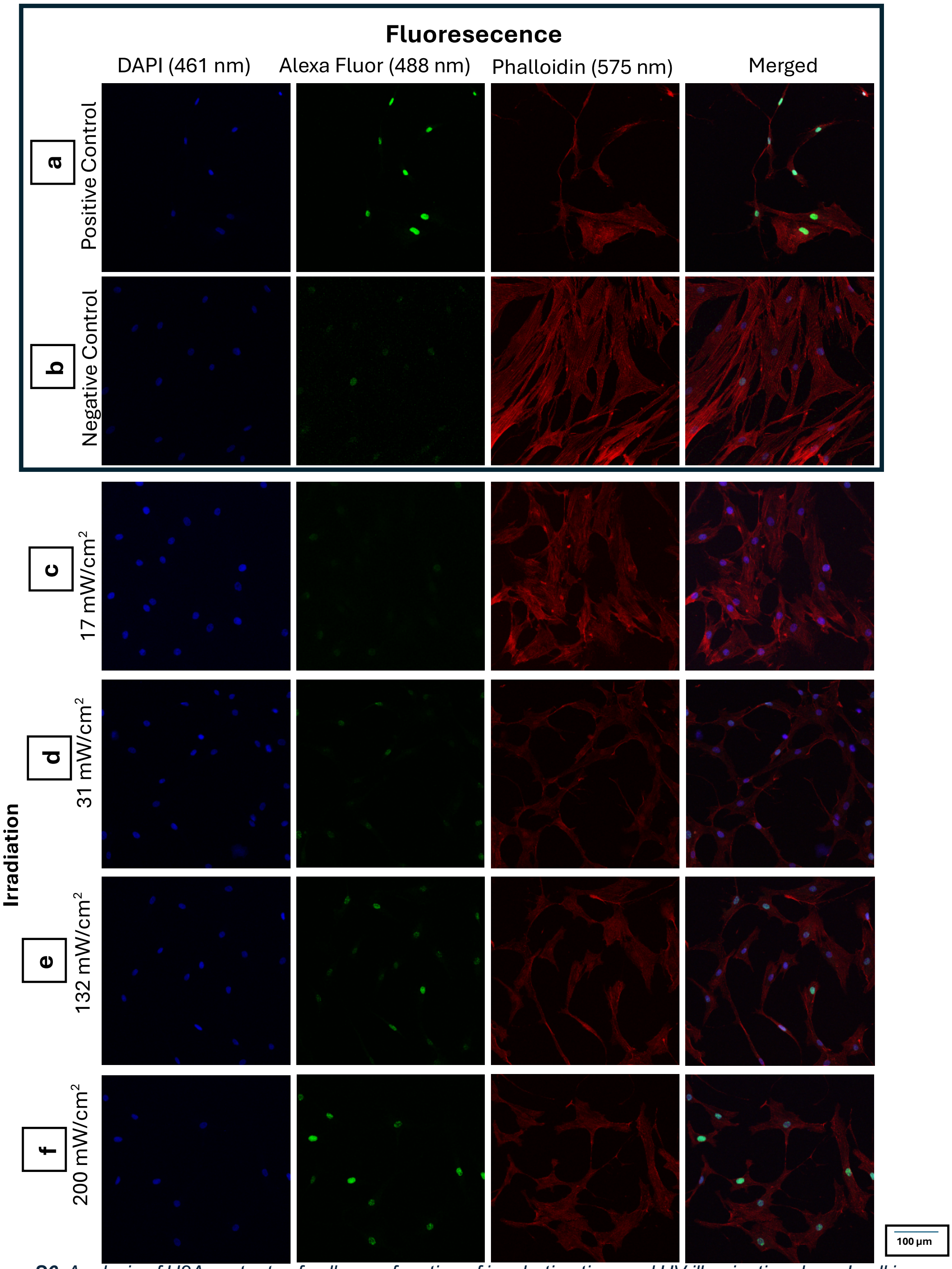

Figure S6. *Analysis of H2A contents of cells as a function of incubation time and UV illumination dose. In all images, the red color represents the F actin cytoskeleton stained with Phalloidin. The bright green fluorescence marks the presence of H2A, with its intensity serving as a quantitative indicator of H2A release and DAPI indicates the cell´s nuclei with blue color. Control /reference for H2A presence including a positive control (a), where cells were subjected to a high UV dose of 200 mW/cm² for 10 min and (b) a negative control with no UV treatment. (c), (d) (e) and (f),: experimental results in rows, representing various UV illumination doses applied to the cells for 180 sec. The green fluorescence in each image reflects the amount of H2A released, under each condition after incubation. The results indicate that while cells exhibit an initial response to UV induced environmental changes, characterized by H2A release, they show significant decrease in release H2A in the HTF cells nuclei at 1 hr only. The cell staining experiment utilizing H2A, DAPI, and Phalloidin stains as indicators of UV illumination was conducted in triplicate across multiple experimental runs, achieving consistent success in all cases.*

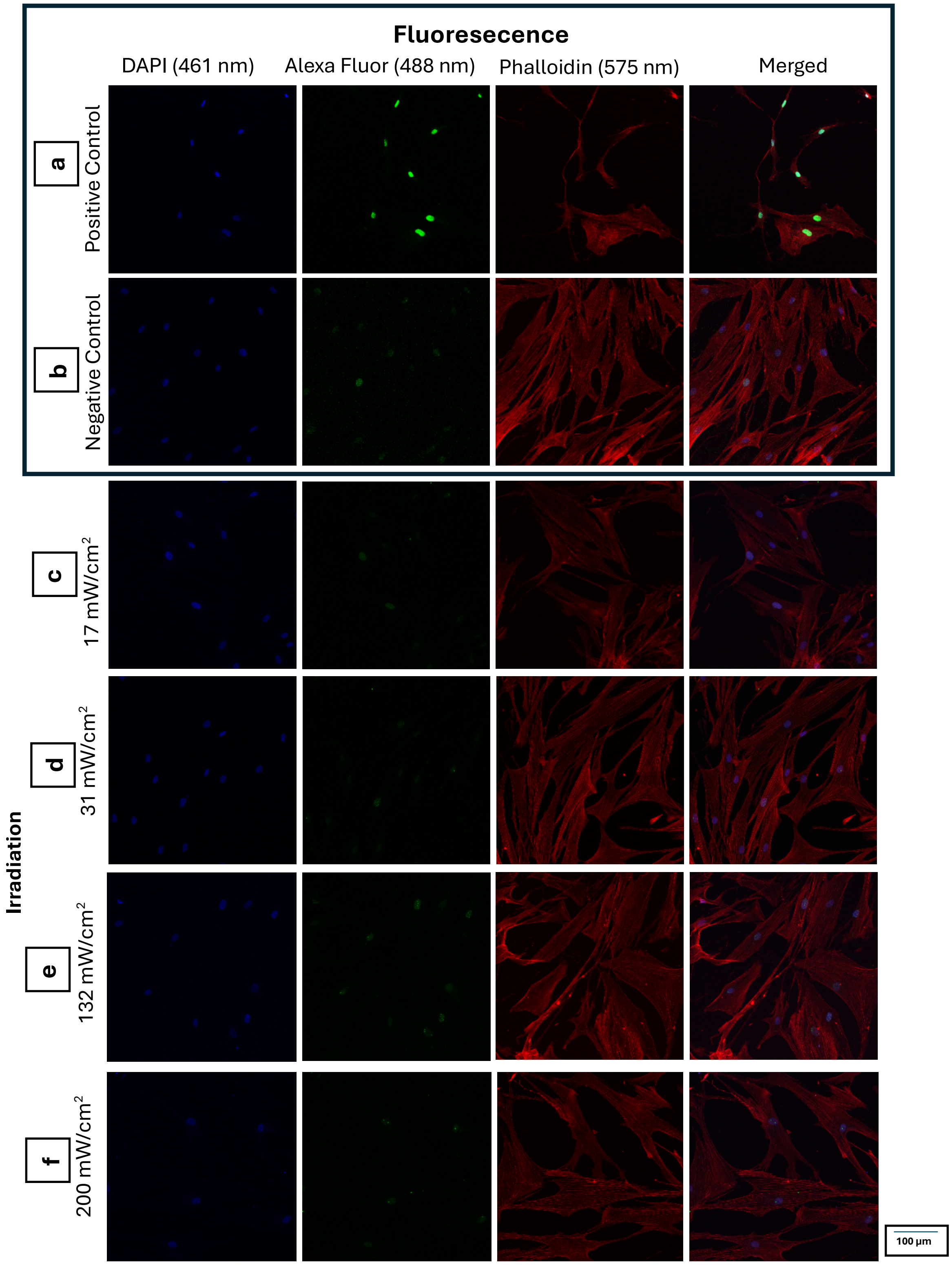

Figure S7. *Analysis of H2A contents of cells as a function of incubation time and UV illumination dose. In all images, the red color represents the F actin cytoskeleton stained with Phalloidin. The bright green fluorescence marks the presence of H2A, with its intensity serving as a quantitative indicator of H2A release and DAPI indicates the cell´s nuclei with blue color. Control /reference for H2A presence including a positive control (a), where cells were subjected to a high UV dose of 200 mW/cm² for 10 min and (b) a negative control with no UV treatment. (c), (d) (e) and (f),: experimental results in rows, representing various UV illumination doses applied to the cells for 180 sec. The green fluorescence in each image reflects the amount of H2A released, under each condition after incubation. The results indicate that while cells exhibit an initial response to UV induced environmental changes, characterized by H2A release, they show significant decrease in release H2A in the HTF cells nuclei at 48 hrs. The cell staining experiment utilizing H2A, DAPI, and Phalloidin stains as indicators of UV illumination was conducted in triplicate across multiple experimental runs, achieving consistent success in all cases.*

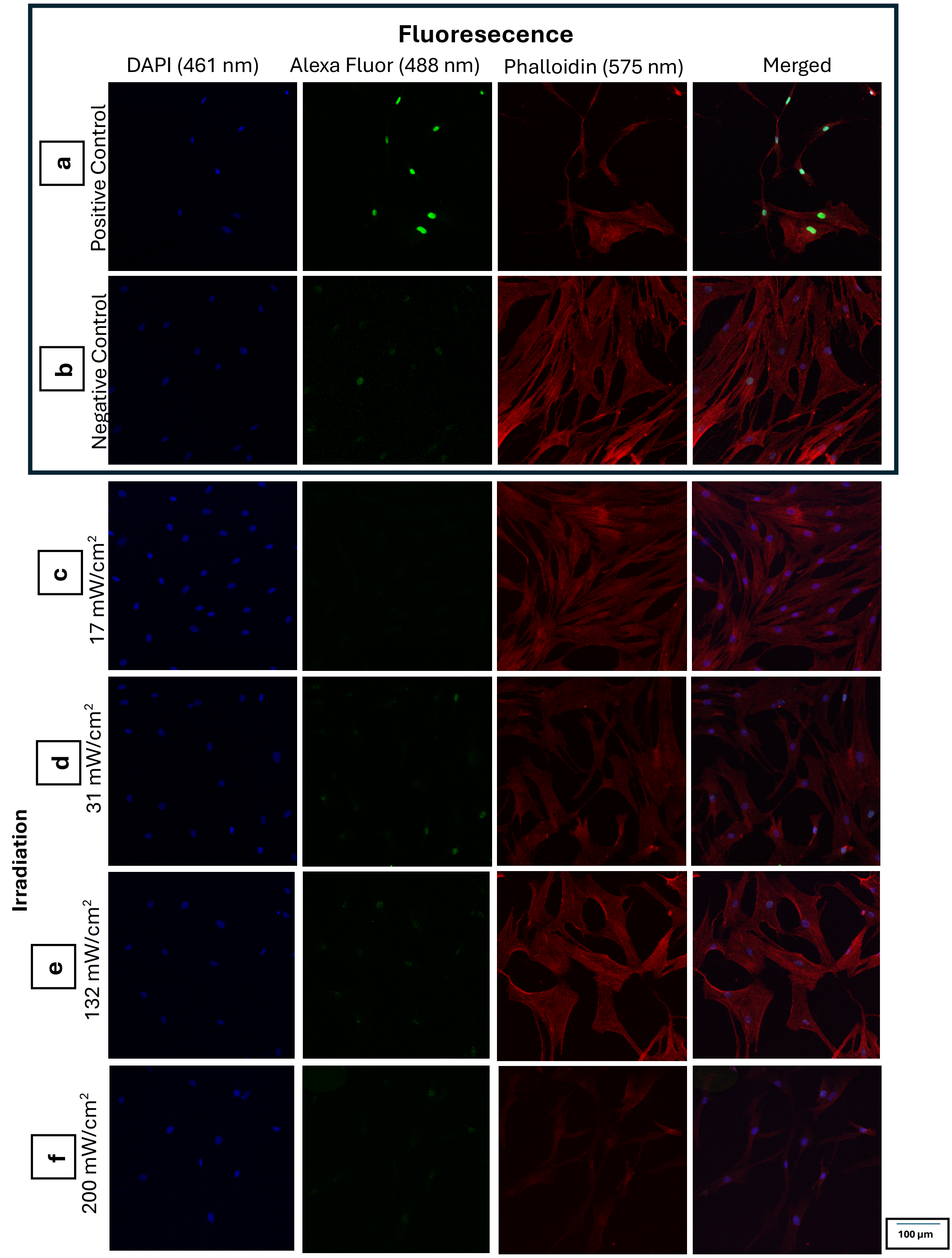

Figure S8. *Analysis of H2A contents of cells as a function of incubation time and UV illumination dose. In all images, the red color represents the F actin cytoskeleton stained with Phalloidin. The bright green fluorescence marks the presence of H2A, with its intensity serving as a quantitative indicator of H2A release and DAPI indicates the cell´s nuclei with blue color. Control /reference for H2A presence including a positive control (a), where cells were subjected to a high UV dose of 200 mW/cm² for 10 min and (b) a negative control with no UV treatment. (c), (d) (e) and (f),: experimental results in rows, representing various UV illumination doses applied to the cells for 180 sec. The green fluorescence in each image reflects the amount of H2A released, under each condition after incubation. The results indicate that while cells exhibit an initial response to UV induced environmental changes, characterized by H2A release, they show significant decrease in release H2A in the HTF cells nuclei at 7 days. The cell staining experiment utilizing H2A, DAPI, and Phalloidin stains as indicators of UV illumination was conducted in triplicate across multiple experimental runs, achieving consistent success in all cases.*

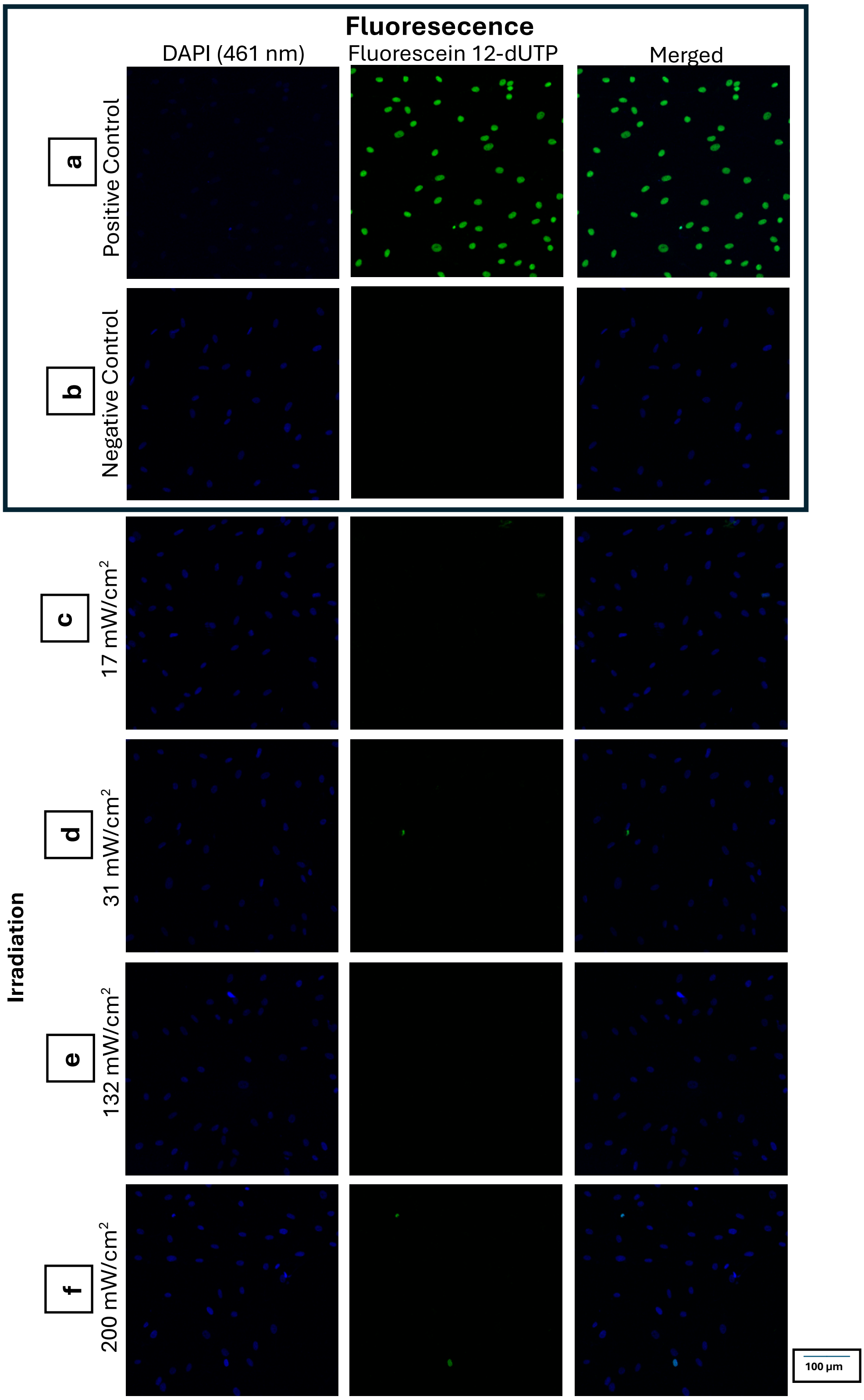

Figure S9. *Fluorescence imaging cells after UV illumination with various doses and addition of DAPI and fluorescein 12-dUTP as fluorescence indicator in an apoptosis test. Apoptotic cells are indicated by green spots because of fluorescence indicator Fluorescein 12-dUTP. (a)Positive control after treating the cells with DNAse, an enzyme for degrading DNA to fragmentation. (b) Negative control with the same process, but no UV irradiation or DNAse. (c), (d), (e), and (f): These panels display the results of cells exposed to UV light at varying energy doses. The absence of green fluorescence in each image indicates no presence of apoptotic cells. The cell density was 10,000 cells per well in a 24-well plate. The cell staining experiments, using 12-dUTP, and DAPI stains as markers of UV-induced damage, were performed three times during multiple experimental runs (more than five) and consistently yielded successful results. Strict adherence to the established protocol ensured reliable and error-free outcomes.*

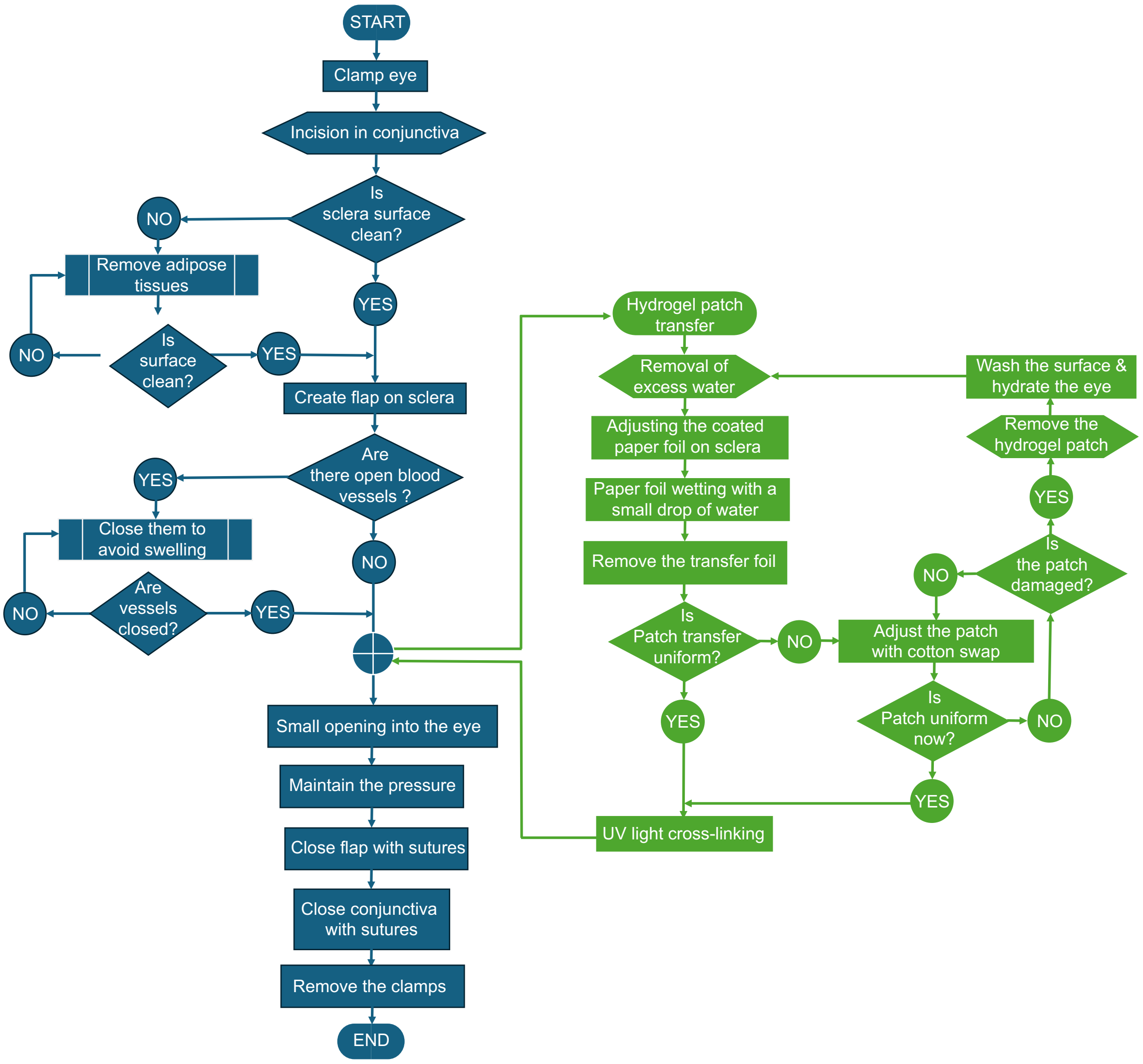

Figure S10. *Schematic flowchart illustrating the procedural steps of a trabeculectomy surgery. The steps depicted in blue represent the conventional surgical protocol, while the green pathway highlights the integration of a protein-repellent hydrogel application. In this modified approach, the hydrogel patch is transferred prior to creating the scleral opening to avoid complications from exposure to aqueous humor flow. The hydrogel patch is successfully transferred and cross-linked onto the sclera within 4-5 minutes, a minimal extension to the overall surgery duration. This advanced step ensures a controlled and efficient integration without significant delays in the procedure.*

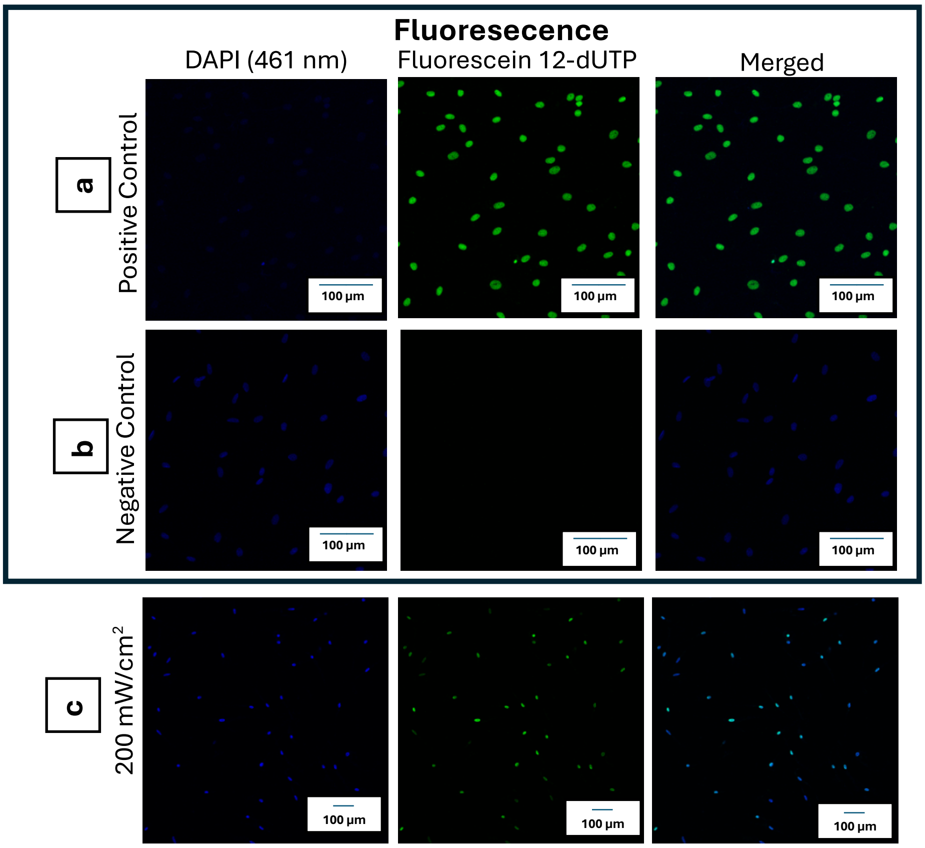

Figure S11. *Fluorescence imaging cells after UV illumination with various doses and addition of DAPI and fluorescein 12-dUTP as fluorescence indicator in an apoptosis test. Apoptotic cells are indicated by green spots because of fluorescence indicator Fluorescein 12-dUTP. (a)Positive control after treating the cells with DNAse, an enzyme for degrading DNA to fragmentation. (b) Negative control with the same process, but no UV irradiation or DNAse* (c) it shows that cells exposed to UV light for an extended duration of 10 min with the dose of 200 mW/cm² indicating green fluorescence provides insight into the cumulative effect of prolonged UV exposure on inducing apoptosis in HTF cells. *The cell density was 10,000 cells per well in a 24-well plate. The cell staining experiments, using 12-dUTP, and DAPI stains as markers of UV-induced damage, were performed three times during multiple experimental runs (more than five) and consistently yielded successful results. Strict adherence to the established protocol ensured reliable and error-free outcomes.*

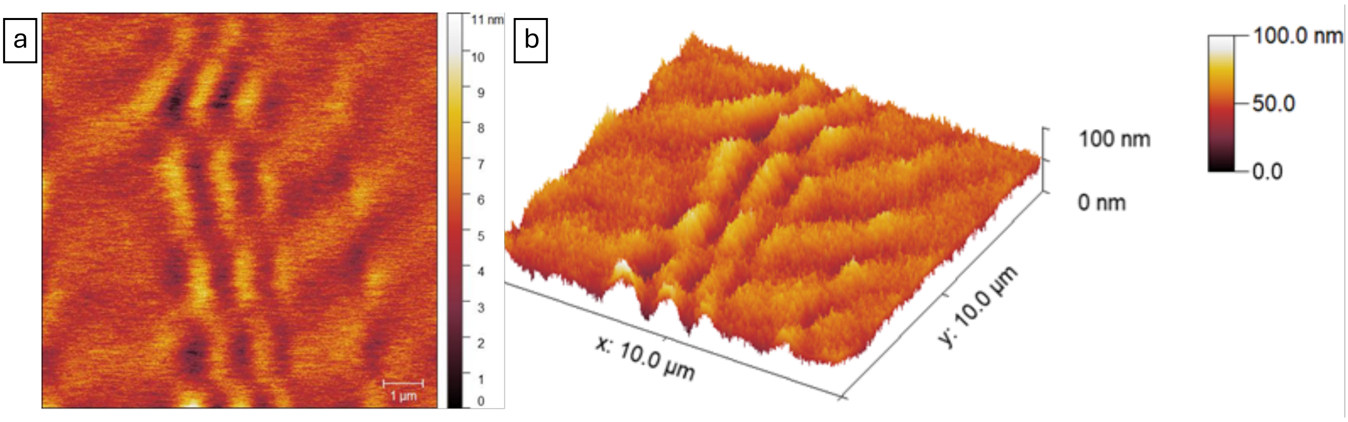

***Figure S12**. Atomic force microscopy (AFM) images of the hydrogel patch transferred onto a glass slide using a flexible transfer foil with a tiny drop of water as the release mechanism. (a) A two-dimensional (2D) AFM scan of a 10 × 10 µm area collected in AC mode using the JPK NanoWizard® 4 XP NanoScience system. The histogram on the right indicates vertical surface non-uniformity in nanometers (nm), where darker regions represent areas with nearly 0 nm thickness, and lighter regions indicate increasing surface roughness. (b) A three-dimensional (3D) visualization of the same hydrogel patch shows the X and Y axes (10 µm each) with the corresponding vertical height. The surface roughness observed is likely due to the retraction of the hydrogel on the hydrophobic glass slide. Imaging on the sclera with the patch attached was not feasible due to its highly irregular and rough surface in both wet and dry states. The AFM measurements were performed using a Budget Sensors™ Tap190-G cantilever with a rotated shape (height: 15–19 µm, setback: 10–20 µm, tip radius: 10 nm) and half-cone angles of 20–25° along the cantilever, 25–30° from the side, and 10° at the apex. A general correction factor of 0.81 was applied, with 0.251 used when the first resonance frequency was too low.*
